# Overview of PacELF—the Pacific Programme for the Elimination of Lymphatic Filariasis

**DOI:** 10.1186/s41182-017-0075-4

**Published:** 2017-11-01

**Authors:** Kazuyo Ichimori, Patricia M. Graves

**Affiliations:** 1 Institute of Tropical Medicine, Nagasaki University, Nagasaki, 852-8523 Japan; 2 College of Public Health, Medical and Veterinary Sciences, Division of Tropical Health and Medicine, James Cook University, Cairns, Queensland 4870 Australia

This special issue of *Tropical Medicine and Health* has been produced by the PacELF Endgame project to record and celebrate the successes of PacELF—the Pacific Programme for the Elimination of Lymphatic Filariasis.

Lymphatic filariasis (LF) is a nematode worm-caused infection transmitted by mosquitoes. Male and female adult worms develop in humans from larvae injected by mosquitoes of several genera. The worms lodge in the lymph vessels and nodes, where they mate and live for several years, producing microfilariae that circulate in the peripheral blood and can be ingested by mosquitoes to continue the cycle. Worms in the lymphatic system contribute to acute attacks of dermatolymphangitis caused by secondary bacterial infection or lymphangitis arising from death of worms. Resulting chronic morbidity can be severe, irreversible, and lifelong, including enlarged limbs (lymphoedema and elephantiasis) and hydrocoele (swollen scrotum in men). Filariasis can be treated with deworming drugs, which are effective at resolving the infection at an early stage, but chronic disabling consequences are harder to address. Hydrocoele can be resolved by surgery. Interrupting transmission requires widespread repeated treatment of mostly asymptomatic people (who may have microfilariae in their blood) coupled with vector control.

Lymphatic filariasis has long been a highly endemic scourge in the Pacific, with infection rates amongst the highest in the world. In this area, all LF is caused by the species *Wuchereria bancrofti*, with different ecologies based on the local mosquito vectors (*Anopheles*, *Culex*, or *Aedes*) and the periodicity (time when microfilariae are at highest density in the blood).

Both Japan and Australia had succeeded in interrupting transmission of LF by the 1970s. Several Pacific countries had been working more or less independently on LF control. In Fiji, the late Dr. Mataika and his staff made substantial progress in mapping and instigating programs in areas of that country, while in Samoa, the Ministry of Health supported by WHO conducted annual mass drug administration with different drug regimens. Mass drug administration was also conducted to a limited extent in other countries, including American Samoa and Vanuatu. In French Polynesia, the Ministry of Health and Institut Malardé, as a pioneer institute of Pacific filariasis, had also been very active in LF control and studies.

Despite these excellent efforts, there had really been no coordinated global or regional plans to control LF. Then, in 1993, the International Task Force for Disease Eradication identified lymphatic filariasis as one of only six eradicable or potentially eradicable diseases. This conclusion was based on newly available, safe, and cost-effective control methods, including improved diagnostic tools, improved drugs and drug combinations, mosquito control, and effective strategies for dealing with end-stage disease. In the 1990s, a number of events and activities brought LF to a higher profile. The scene was set for a global effort.

In May 1997, the World Health Assembly (WHA) passed a resolution urging Member States “to strengthen activities toward eliminating lymphatic filariasis as a public health problem, and requesting the Director-General to mobilize support for global and national elimination activities.” (WHA Resolution 50.29). These events served to increase the awareness of the disease as a public health problem and alerted Pacific Island countries and territories of the need to control or eliminate filariasis. In the Pacific region, support had already been building through two international meetings on lymphatic filariasis elimination convened by WHO and James Cook University (JCU): one in Bali, Indonesia, in 1996 and one in Townsville, Australia, in July 1997 coinciding with the official opening of the JCU WHO Collaborating Centre on Control of Lymphatic Filariasis.

In March 1999, a meeting of the Secretariat of the Pacific Community (SPC) was held in Palau in coordination with the WHO Pacific Ministers of Health meeting. The SPC Heads of Health Services Consultative Meeting took up this call to action and reported in their resulting statement on Healthy Islands: “In keeping with the WHA resolution to eliminate lymphatic filariasis, the meeting encouraged the Secretariat to continue discussions with WHO, and other relevant donor agencies to develop and implement a comprehensive strategy to eliminate lymphatic filariasis in all 22 island countries and territories.” At this time, both WHO and SPC were well positioned to work together on LF control. WHO had vector-borne disease and communicable diseases experts in Manila, Papua New Guinea (PNG), Solomon Islands, and Vanuatu. The SPC was implementing the Pacific Regional Vector Borne Diseases Project and had staff based at SPC HQ in Noumea, New Caledonia, and project offices in Vanuatu, Solomon Islands, and Fiji.

Dr. Kazuyo Ichimori from WHO and Dr. Tony Stewart from SPC subsequently met in Port Vila, Vanuatu, to discuss how best to enact the resolutions of the WHA in the region and the regional program initiatives discussed in Palau in March 1999. Funding was secured from WHO and through SPC’s vector-borne diseases project to hold a meeting of Pacific countries in Brisbane on the 28th and 29th June 1999. Participants were public health leaders from those Pacific countries with recent or current transmission of lymphatic filariasis, together with staff from SPC, WHO, and other institutions working in the field of elimination of filariasis. This meeting provided a forum for the country representatives from American Samoa, Cook Islands, Fiji, French Polynesia, Nauru, New Caledonia, Niue, PNG, Samoa, Solomon Islands, Tonga, Tuvalu, Vanuatu, and Wallis and Futuna to discuss current and planned activities globally and within the Pacific region. Representatives from University of Queensland, James Cook University, SmithKline Beecham, and AMRAD ICT also participated. The 14 countries attending the meeting refined and endorsed a regional plan of action, and appointed four country representatives to form the interim coordinating body, in order to facilitate implementation between meetings. Thus, the world’s first regional LF control program—PacELF—was born.

Pacific countries and territories represent a subset of countries in the WHO WPRO region. PacELF was designed as a regional program driven by the countries and constituted a network of the 22 island countries and territories in the Pacific for the sole purpose of eliminating filariasis in the Pacific. The strategy for achieving this goal was annual mass drug administration (MDA) using diethylcarbamazine (DEC) with albendazole to stop transmission, together with clinical management of infections to minimize progression of pathology in individuals already infected. Since 1999, PacELF has been supported by many partners including WHO, WPRO, Government of Japan, JICA, AusAID (now DFAT), UK VSO, USAID, New Zealand, Republic of Korea, the NTD support center at the Task Force for Global Health, James Cook University, Nagasaki University, the Centers for Disease Control and Prevention, and GlaxoSmithKline.

At the start of PacELF, 16 of the 22 Pacific countries and territories were classified as LF endemic through having prevalence > 1% in at least part of the country. Mass drug administration (MDA) has been conducted in 15 of these countries (excluding New Caledonia due to uncertainty about the need for MDA), with the first starting in 1999. Over the next 18 years, under the leadership of four different health officers from the WHO Western Pacific Regional Office [see Table 1], the program addressed a number of critical issues—often requiring pioneering solutions to problems being faced for the first time in any of the GPELF countries. These included monitoring strategies using sentinel site and spot check surveys, the routine use of antigen rather than microfilaria tests, and large population-based surveys in all ages designed to determine whether MDA should be stopped. Many countries stopped MDA in the mid- to late 2000s, although five are continuing to the present day. The strategies used, monitoring and elimination guidelines, and status of progress towards elimination up to 2005 were described in “The PacELF Way,” a book published in 2006 (http://iris.wpro.who.int/handle/10665.1/10966). The endemicity status of countries before PacELF, in 2000 and in 2017, can be seen in Maps 1, 2, and 3.

**Table 1:** PacELF leadership at WHO Division of Pacific Technical Support, Suva, Fiji, showing major strategies and achievements, 1999 to 2017

1999 to 2005: Dr. Kazuyo Ichimori	
• Baseline surveys conducted using ICT	
• Established a coordinating body and PacELF office and warehouse	
• Established logistics systems for drugs, diagnostics and data reporting	
• Regular program managers’ meetings	
• Development of M&E plan using A (baseline), B (sentinel), C (stop MDA), and D (transmission) surveys; C survey was in all ages and D survey in children	
• First countries stopped MDA in 2004	
• Initiated morbidity surveys	
• Published PacELF book	
2006 to 2008: Dr. Corinne Capuano	
• M&E guideline revision—maintained stop MDA (C) surveys in all ages and transmission surveys in children, with mop up around positive cases	
• Introduced test and treat strategies in three countries	
• Improvements to behavior change communication	
• Morbidity surveys and hydrocoele surgery programs in Fiji	
• Maintained MDA and post-MDA surveillance	
2009 to 2011: Dr. Sunghye Kim	
• Preparation of elimination dossiers	
• Maintained MDA and post-MDA surveillance	
2012 to 2017: Dr. Padmasiri Aratchige	
• Maintained MDA as needed and oversaw transmission assessment surveys	
• Validation of elimination dossiers approved for Vanuatu, Republic of Marshall Islands, Niue, Cook Is, and Tonga

**Map 1:**
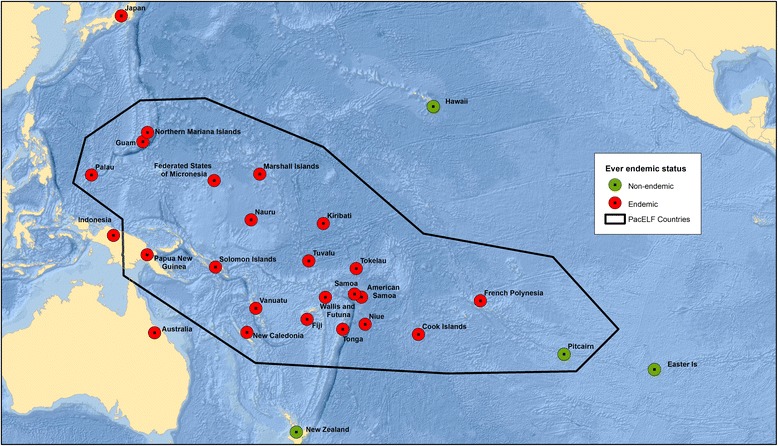
LF in the Pacific—countries and territories ever endemic

**Map 2:**
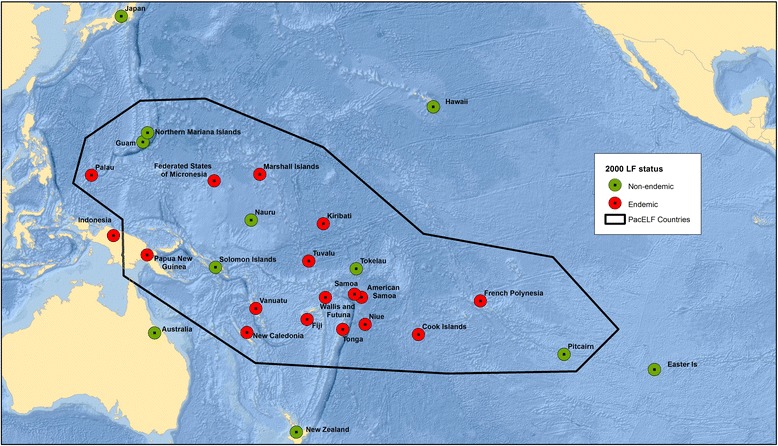
LF in the Pacific—countries and territories endemic in 2000

**Map 3:**
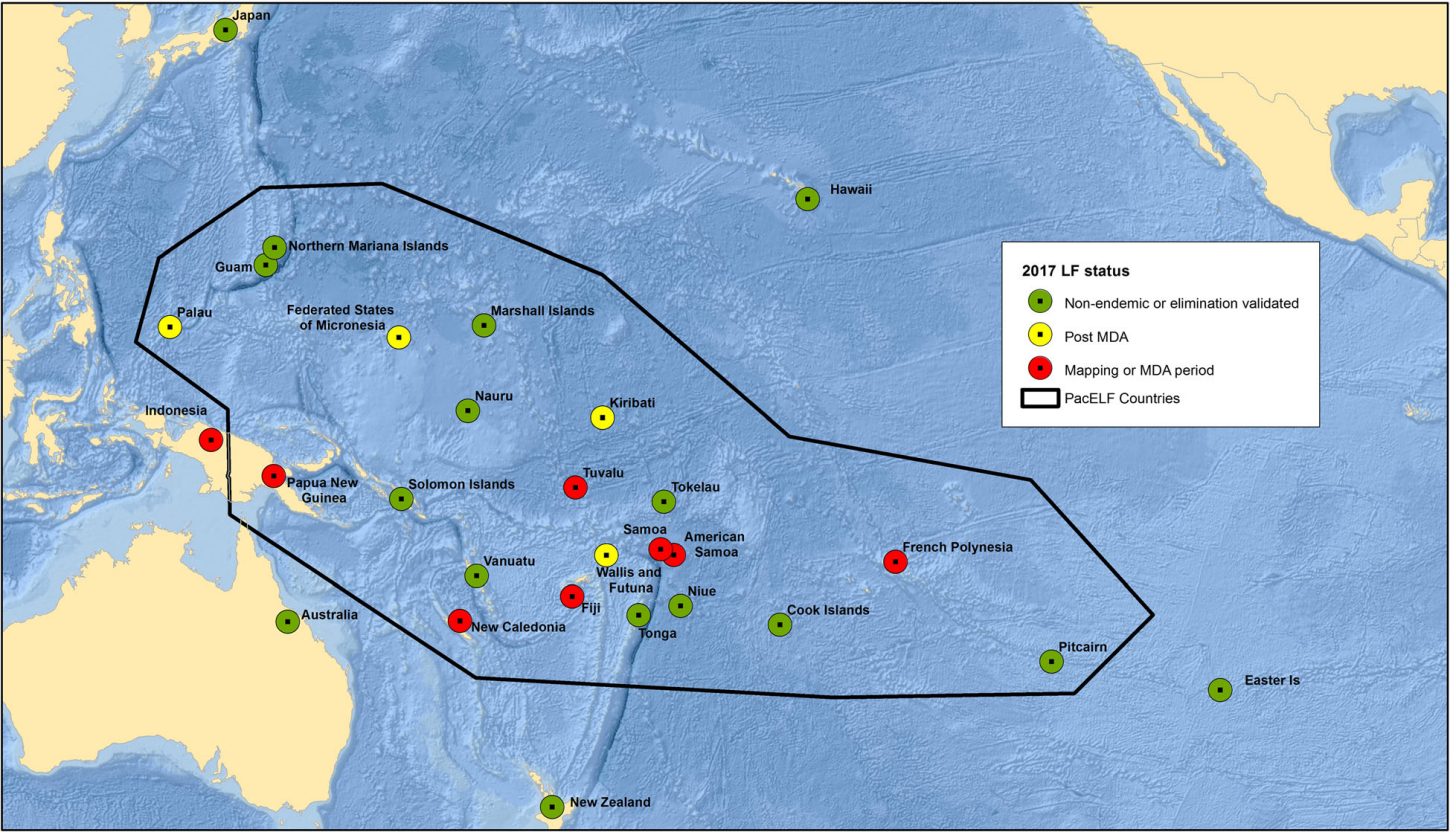
LF in the Pacific—countries and territories endemic in 2017

Those countries that reduced prevalence low enough to stop MDA then entered several years of post-MDA surveillance during which they conducted further surveys to validate elimination. These surveys followed a series of guidelines produced by PacELF and the GPELF that evolved over the years. The initial PacELF monitoring and evaluation strategy was developed in 2003, modified in 2008 to include innovative test and treat and contact tracing strategies, and was coordinated with the global strategy using “Transmission Assessment Surveys” by 2011 (http://apps.who.int/iris/handle/10665/44580).

For validation, it is required that each country produce a “dossier” to present the evidence to WHO that LF has been eliminated as a public health problem. The following countries have received validation of their dossiers by 2017: Vanuatu, Niue, Cook Islands, Republic of the Marshall Islands, and Tonga. Several others are close to submitting dossiers. Problems and challenges for the other countries include the need to rapidly and completely scale-up interventions, the possibility of resurgence from remaining LF hotspots of transmission, and the difficulty of ensuring that those with residual chronic morbidity are provided with quality services.

To celebrate PacELF and the countries’ achievements in LF elimination, this special issue reports on the progress towards LF elimination in selected countries. We present here maps showing the status of the countries in the region at three time points: (1) those that were ever endemic, (2) endemicity at the start of the global program (2000), and (3) the status in 2016. The countries described in this issue include one with highly successful and rapid progression to validation of LF elimination as a public health problem (Vanuatu) as well as those with lingering challenges (Federated States of Micronesia and American Samoa). Papers from other countries will be included as elimination is achieved and their dossiers become available. We take the opportunity also to provide a comprehensive bibliography of published papers on LF in the Pacific (including Australia and Japan) for future reference (see Additional file 1).

We hope that these success stories will be both a source of inspiration to the other regions of the world also engaged in such effort and a source of justifiable pride to all the countries and territories in the Pacific.

## Additional file

Additional file 1: Bibliography of published literature on lymphatic filariasis in the Pacific, Australia, and Japan, 1970 to July 2017. (DOCX 91 kb)

