# Supplementary material for: Overview of PacELF—the Pacific Programme for the Elimination of Lymphatic Filariasis
Source: Trop Med Health. 2017 Nov 1;45:34. doi: 10.1186/s41182-017-0075-4 (PMC5664808; doi:10.1186/s41182-017-0075-4)
Supplement: Additional file 1: — Bibliography of published literature on lymphatic filariasis in the Pacific, Australia, and Japan, 1970 to July 2017. (DOCX 91 kb) [file 41182_2017_75_MOESM1_ESM.docx]

**Bibliography of published literature on Lymphatic Filariasis in the Pacific, Australia and Japan,
1970 to July 2017.**

To provide full background on lymphatic filariasis in the Pacific, a bibliography of published literature is provided here. We conducted a systematic search in PubMed for studies of lymphatic filariasis up to 27 July 2017 using the terms [‘filar*’ or ‘Bancroft*’] and [Oceania or Pacific or (each individual country name)]] in the title, abstract or keywords. All PacELF countries and territories (see overview) plus the formerly endemic Australia and Japan were included. We then restricted the search to documents published in 1970 or later. Additional publications known to the authors were added.

Studies were included if they contained information on filariasis in the region (or its vectors) and were published in peer reviewed literature, or in books or reports that are publicly available.

Studies were excluded if they were not about human filariasis or were from areas outside the Pacific, Australia and Japan (with the exception of imported cases). Studies solely describing preclinical laboratory studies or disease modelling were excluded unless of very direct relevance to the Pacific.

This list builds on previous literature summarised in several reviews.

The Secretariat for the Pacific Community (then called South Pacific Commission) published a series of annotated bibliographies between 1954 and 1960. The most comprehensive one is:
Iyengar MOT (1959). A review of the literature on the distribution and epidemiology of filariasis in the South Pacific Region. SPC Technical Report No 126. Noumea, New Caledonia.

Sasa (1976) published a comprehensive global review of lymphatic filariasis:
Sasa M. Human Filariasis. A Global Survey of Epidemiology and Control. Baltimore. London. Tokyo: University Park Press; 1976.

Melrose (2004) published a global history of lymphatic filariasis from 1862 to 2002 with extensive cited literature:
Melrose W. Lymphatic filariasis: a review 1862-2002. Killarney, Qld: Warwick Educational Publishing Inc, Secretariat, 29 Kurrajong St., Killarney Qld 4373, Australia; 2004.

The PacELF Way book includes references for the Pacific Island countries and territories up to 2005:
WHO (2006). The PacELF Way: Towards the elimination of lymphatic filariasis from the Pacific, 1999-2005. Manila: WHO Western Pacific Region; 2006.

1. Abe M, Yaviong J, Taleo G, Ichimori K. Microfilarial periodicity of *Wuchereria bancrofti* in Vanuatu. Transactions of the Royal Society of Tropical Medicine and Hygiene. 2003;97(5):498-500.
2. Addiss DG. Mass treatment of filariasis in New Guinea. New England Journal of Medicine. 2003;348(12):1179-80.
3. Afifi SE, Spencer M, Hudson PB, Tavil NW. Biting prevalence and malaria transmission patterns in the *Anopheles punctulatus* complex (Diptera: Culicidae) in Papua New Guinea. Australian Journal of Experimental Biology and Medical Science. 1980;58(1):1-17.
4. Alexander N, Moyeed R, Stander J. Spatial modelling of individual-level parasite counts using the negative binomial distribution. Biostatistics. 2000;1(4):453-63.
5. Alexander ND. *Wuchereria bancrofti* infection and disease in a rural area of Papua New Guinea. Papua New Guinea Medical Journal. 2000;43(3-4):166-71.
6. Alexander ND, Moyeed RA, Hyun PJ, Dimber ZB, Bockarie MJ, Stander J, et al. Spatial variation of *Anopheles*-transmitted *Wuchereria bancrofti* and *Plasmodium falciparum* infection densities in Papua New Guinea. Filaria Journal. 2003;2(1):14.
7. Alexander NDE, Bockarie MJ, Dimber ZB, Griffin L, Kazura JW, Alpers MP. Migration and dispersal of lymphatic filariasis in Papua New Guinea. Transactions of the Royal Society of Tropical Medicine and Hygiene. 2001;95(3):277-9.
8. Alexander NDE, Bockarie MJ, Kastens WA, Kazura JW, Alpers MP. Absence of ivermectin-associated excess deaths. Transactions of the Royal Society of Tropical Medicine and Hygiene. 1998;92(3):342-.
9. Alexander NDE, Grenfell BT. The effect of pregnancy on *Wuchereria bancrofti* microfilarial load in humans. Parasitology. 1999;119:151-6.
10. Alexander NDE, Kazura JW, Bockarie MJ, Perry RT, Dimber ZB, Grenfell BT, et al. Parental infection confounded with local infection intensity as risk factors for childhood microfilaraemia in bancroftian filariasis. Transactions of the Royal Society of Tropical Medicine and Hygiene. 1998;92(1):23-4.
11. Alexander NDE, Perry RT, Dimber ZB, Hyun PJ, Alpers MP, Kazura JW. Acute disease episodes in a *Wuchereria bancrofti*-endemic area of Papua New Guinea. American Journal of Tropical Medicine and Hygiene. 1999;61(2):319-24.
12. Aliota MT, Chen CC, Dagoro H, Fuchs JF, Christensen BM. Filarial worms reduce *Plasmodium* infectivity in mosquitoes. PLoS Neglected Tropical Diseases. 2011;5(2):e963.
13. Allen T, Taleo F, Graves PM, Wood P, Taleo G, Baker MC, et al. Impact of the Lymphatic Filariasis Control Program towards elimination of filariasis in Vanuatu, 1997-2006. Tropical Medicine and Health. 2017;45:8.
14. Andrews JR. The parasitology of the Maori in pre-European times. New Zealand Medical Journal. 1976;84(568):62-5.
15. Anon. Lymphatic filariasis in Fiji: Incidence and review of the Literature. Fiji Journal of Public Health. 2012;1(1):16-8.
16. Aoki Y, Fujimaki Y, Tada I. Basic studies on filaria and filariasis. Tropical Medicine and Health. 2011;39(1 Suppl 2):51-5.
17. Arnold BF, van der Laan MJ, Hubbard AE, Steel C, Kubofcik J, Hamlin KL, et al. Measuring changes in transmission of neglected tropical diseases, malaria, and enteric pathogens from quantitative antibody levels. PLoS Neglected Tropical Diseases. 2017;11(5):e0005616.
18. Ashford RW, Babona D. The parasites of the Purari people of Gulf Province, Papua New Guinea. Papua New Guinea Medical Journal. 1980;23(4):165-8.
19. Attenborough RD, Burkot TR, Gardner DS. Altitude and the risk of bites from mosquitoes infected with malaria and filariasis among the Mianmin people of Papua New Guinea. Transactions of the Royal Society of Tropical Medicine and Hygiene. 1997;91(1):8-10.
20. Ayres M, Salzano FM, Helena M, Franco LP, de Souza Barros RM. The association of blood groups, ABH secretion, haptoglobins and hemoglobins with filariasis. Human Heredity. 1976;26(2):105-9.
21. Beebe NW, Cooper RD. Distribution and evolution of the *Anopheles punctulatus* group (Diptera: Culicidae) in Australia and Papua New Guinea. International Journal of Parasitology. 2002;32(5):563-74.
22. Beebe NW, Ellis JT, Cooper RD, Saul A. DNA sequence analysis of the ribosomal DNA ITS2 region for the *Anopheles punctulatus* group of mosquitoes. Insect Molecular Biology. 1999;8(3):381-90.
23. Beebe NW, Maung J, van den Hurk AF, Ellis JT, Cooper RD. Ribosomal DNA spacer genotypes of the *Anopheles bancroftii* group (Diptera: Culicidae) from Australia and Papua New Guinea. Insect Molecular Biology. 2001;10(5):407-13.
24. Beebe NW, Russell T, Burkot TR, Cooper RD. *Anopheles punctulatus* group: evolution, distribution, and control. Annual Review of Entomology. 2015;60:335-50.
25. Behbahani A, Dutton TJ, Davies N, Townson H, Sinkins SP. Population differentiation and *Wolbachia* phylogeny in mosquitoes of the *Aedes scutellaris* group. Medical and Veterinary Entomology. 2005;19(1):66-71.
26. Behbahani A, Dutton TJ, Raju AK, Townson H, Sinkins P. Polymorphic microsatellite loci in the mosquito *Aedes polynesiensis*. Molecular Ecology Notes. 2004;4(1):59-61.
27. Belizario V, Jr., Delos Trinos JP, Garcia NB, Reyes M. Cutaneous manifestations of selected parasitic infections in Western Pacific and Southeast Asian Regions. Current Infectious Disease Reports. 2016;18(9):30.
28. Bockarie M. Can lymphatic filariasis be eradicated in Papua New Guinea. Papua New Guinea Medical Journal. 1994;37(2):61-4.
29. Bockarie M, Kazura J, Alexander N, Dagoro H, Bockarie F, Perry R, et al. Transmission dynamics of *Wuchereria bancrofti* in East Sepik Province, Papua New Guinea. American Journal of Tropical Medicine and Hygiene. 1996;54(6):577-81.
30. Bockarie MJ. Molecular xenomonitoring of lymphatic filariasis. American Journal of Tropical Medicine and Hygiene. 2007;77(4):591-2.
31. Bockarie MJ, Alexander N, Bockarie F, Ibam E, Barnish G, Alpers M. The late biting habit of parous *Anopheles* mosquitoes and pre-bedtime exposure of humans to infective female mosquitoes. Transactions of the Royal Society of Tropical Medicine and Hygiene. 1996;90(1):23-5.
32. Bockarie MJ, Alexander NDE, Hyun P, Dimber Z, Bockarie F, Ibam E, et al. Randomised community-based trial of annual single-dose diethylcarbamazine with or without ivermectin against *Wuchereria bancrofti* infection in human beings and mosquitoes. Lancet. 1998;351(9097):162-8.
33. Bockarie MJ, Fischer P, Williams SA, Zimmerman PA, Griffin L, Alpers MP, et al. Application of a polymerase chain reaction-ELISA to detect *Wuchereria bancrofti* in pools of wild-caught *Anopheles punctulatus* in a filariasis control area in Papua New Guinea. American Journal of Tropical Medicine and Hygiene. 2000;62(3):363-7.
34. Bockarie MJ, Hii JLK, Alexander NDE, Bockarie F, Dagoro H, Kazura JW, et al. Mass treatment with ivermectin for filariasis control in Papua New Guinea: impact on mosquito survival. Medical and Veterinary Entomology. 1999;13(2):120-3.
35. Bockarie MJ, Ibam E, Alexander ND, Hyun P, Dimber Z, Bockarie F, et al. Towards eliminating lymphatic filariasis in Papua New Guinea: impact of annual single-dose mass treatment on transmission of *Wuchereria bancrofti* in East Sepik Province. Papua New Guinea Medical Journal. 2000;43(3-4):172-82.
36. Bockarie MJ, Jenkins C, Blakie WM, Lagog M, Alpers MP. Control of lymphatic filariasis in a hunter-gatherer group in Madang Province. Papua New Guinea Medical Journal. 2000;43(3-4):196-202.
37. Bockarie MJ, Kazura JW. Lymphatic filariasis in Papua New Guinea: prospects for elimination. Medical Microbiology and Immunology. 2003;192(1):9-14.
38. Bockarie MJ, Tavul L, Ibam I, Kastens W, Hazlett F, Tisch DJ, et al. Efficacy of single-dose diethylcarbamazine compared with diethylcarbamazine combined with albendazole against *Wuchereria bancrofti* infection in Papua New Guinea. American Journal of Tropical Medicine and Hygiene. 2007;76(1):62-6.
39. Bockarie MJ, Tavul L, Kastens W, Michael E, Kazura JW. Impact of untreated bednets on prevalence of *Wuchereria bancrofti* transmitted by *Anopheles farauti* in Papua New Guinea. Medical and Veterinary Entomology. 2002;16(1):116-9.
40. Bockarie MJ, Tisch DJ, Kastens W, Alexander NDE, Dimber Z, Bockarie F, et al. Mass treatment to eliminate filariasis in Papua New Guinea. New England Journal of Medicine. 2002;347(23):1841-8.
41. Boreham PFL, Marks NM. Human filariasis in Australia: introduction, investigation and elimination. Proceedings of the Royal Society of Queensland. 1986;97:23-52.
42. Bouree P, Sauvagnac B, Montaville B. [Lymphatic filariasis in Vanuatu]. Bulletin de la Société de Pathologie Exotique et de ses Filiales. 1987;80(4):634-45.
43. Brabin L. Sex differentials in susceptibility to lymphatic filariasis and implications for maternal child immunity. Epidemiology and Infection. 1990;105(2):335-53.
44. Brelsfoard CL, Dobson SL. *Wolbachia* effects on host fitness and the influence of male aging on cytoplasmic incompatibility in *Aedes polynesiensis* (Diptera: Culicidae). Journal of Medical Entomology. 2011;48(5):1008-15.
45. Brelsfoard CL, Dobson SL. Population genetic structure of *Aedes polynesiensis* in the Society Islands of French Polynesia: implications for control using a *Wolbachia*-based autocidal strategy. Parasites and Vectors. 2012;5:80.
46. Brelsfoard CL, Sechan Y, Dobson SL. Interspecific hybridization yields strategy for South Pacific filariasis vector elimination. PLoSPLoS Neglected Tropical Diseases. 2008;2(1):e129.
47. Bryan JH. Vectors of *Wuchereria bancrofti* in the Sepik Provinces of Papua New Guinea. Transactions of the Royal Society of Tropical Medicine and Hygiene. 1986;80(1):123-31.
48. Bryan JH, Burwell C, Maitland K, Williams T. Culicine mosquitoes (Diptera: Culicidae) attracted to humans on Espiritu Santo, Vanuatu. Medical and Veterinary Entomology. 1996;10(1):101-2.
49. Bryan JH, Dagoro H, Southgate BA. Filarial vector studies in a diethylcarbamazine-treated and in untreated villages in Papua New Guinea. Journal of Tropical Medicine and Hygiene. 1995;98(6):445-51.
50. Bryan JH, Southgate BA. Some observations on filariasis in Western Samoa after mass administration of diethylcarbamazine. Transactions of the Royal Society of Tropical Medicine and Hygiene. 1976;70(1):39-48.
51. Burkot T, Ichimori K. The PacELF programme: will mass drug administration be enough? Trends in Parasitology. 2002;18(3):109-15.
52. Burkot TR, Durrheim DN, Melrose WD, Speare R, Ichimori K. The argument for integrating vector control with multiple drug administration campaigns to ensure elimination of lymphatic filariasis. Filaria Journal. 2006;5:10.
53. Burkot TR, Garner P, Paru R, Dagoro H, Barnes A, McDougall S, et al. Effects of untreated bed nets on the transmission of *Plasmodium falciparum*, *P. vivax* and *Wuchereria bancrofti* in Papua New Guinea. Transactions of the Royal Society of Tropical Medicine and Hygiene. 1990;84(6):773-9.
54. Burkot TR, Handzel T, Schmaedick MA, Tufa J, Roberts JM, Graves PM. Productivity of natural and artificial containers for *Aedes polynesiensis* and *Aedes aegypti* in four American Samoan villages. Medical and Veterinary Entomology. 2007;21(1):22-9.
55. Burkot TR, Molineaux L, Graves PM, Paru R, Battistutta D, Dagoro H, et al. The prevalence of naturally acquired multiple infections of *Wuchereria bancrofti* and human malarias in anophelines. Parasitology. 1990;100 Pt 3:369-75.
56. Burkot TR, Narara A, Paru R, Graves PM, Garner P. Human host selection by anophelines: no evidence for preferential selection of malaria or microfilariae-infected individuals in a hyperendemic area. Parasitology. 1989;98 Pt 3:337-42.
57. Burkot TR, Taleo G, Toeaso V, Ichimori K. Progress towards, and challenges for, the elimination of filariasis from Pacific-island communities. Annals of Tropical Medicine and Parasitology. 2002;96 Suppl 2:S61-9.
58. Cano J, Rebollo MP, Golding N, Pullan RL, Crellen T, Soler A, et al. The global distribution and transmission limits of lymphatic filariasis: past and present. Parasites and Vectors. 2014;7.
59. Cao WC, VanderPloeg CPB, Plaisier AP, vanderSluijs IJS, Habbema JDF. Ivermectin for the chemotherapy of bancroftian filariasis: A meta-analysis of the effect of single treatment. Tropical Medicine and International Health 1997;2(4):393-403.
60. Capuano GP, Capuano C. Surgical management of morbidity due to lymphatic filariasis: the usefulness of a standardized international clinical classification of hydroceles. Tropical Biomedicine. 2012;29(1):24-38.
61. Capuano GP, Capuano C. Reduction of the scrotum by scrotomectomy and plasty in the surgical treatment of large hydroceles in lymphatic filariasis endemic countries. Fiji Journal of Public Health. 2013;2(2):18-25.
62. Capuano GP, Capuano C. Surgical technique: scrotomectomy and plasty of the scrotum in the surgical treatment of large hydroceles in lymphatic filariasis endemic countries. Fiji Journal of Public Health. 2013;2(2):40-4.
63. Carme B. Filarial elephantiasis in French Polynesia: a study concerning the beliefs of 127 patients about the origin of their disease. Transactions of the Royal Society of Tropical Medicine and Hygiene. 1979;73(4):424-6.
64. Carme B, Kaeuffer H, Laigret J. [Eosinophilia and lymphatic filariasis in French Polynesia]. Bulletin de la Société de Pathologie Exotique et de ses Filiales. 1976;69(5):438-45.
65. Carme B, Kaeuffer H, Laigret J, Gentilini M. [Filarial elephantiasis in French Polynesia (*Wuchereria bancrofti* var. *pacifica*). II. Biological aspects]. Bulletin de la Société de Pathologie Exotique et de ses Filiales. 1978;71(6):465-71.
66. Carme B, Laigret J. [Filarial elephantiasis in French Polynesia (*Wuchereria bancrofti* var. *pacifica*). Health study on 274 subjects. I. Epidemiological and Clinical aspects]. Bulletin de la Société de Pathologie Exotique et de ses Filiales. 1978;71(6):455-65.
67. Carme B, Merlin M, Pichon G, Laigret J. Different possibilities for bancroftian filariasis control - theoretical analysis and practical results in French Polynesia. Médecine et Maladies Infectieuses. 1978;8(8):380-4.
68. Cartel JL, Celerier P, Spiegel A, Burucoa C, Roux JF. A single diethylcarbamazine dose for treatment of *Wuchereria bancrofti* carriers in French Polynesia: efficacy and side effects. Southeast Asian Journal of Tropical Medicine and Public Health. 1990;21(3):465-70.
69. Cartel JL, Celerier P, Spiegel A, Plichart R, Roux JF. Effect of two successive annual treatments with single doses of ivermectin on microfilaraemia due to *Wuchereria bancrofti* var. *pacifica*. Transactions of the Royal Society of Tropical Medicine and Hygiene. 1990;84(6):837-9.
70. Cartel JL, Nguyen NL, Spiegel A, Moulia-Pelat JP, Plichart R, Martin PMV, et al. *Wuchereria bancrofti* infection in human and mosquito population of a Polynesian village ten years after interruption of mass chemoprophylaxis with diethylcarbamazine. Transactions of the Royal Society of Tropical Medicine and Hygiene. 1992;86(4):414-6.
71. Cartel JL, Sechan Y, Boutin JP, Celerier P, Plichart R, Roux JF. Ivermectin for treatment of bancroftian filariasis in French Polynesia: efficacy in man, effect on transmission by vector *Aedes polynesiensis*. Tropical Medicine and Parasitology. 1990;41(3):241-4.
72. Cartel JL, Sechan Y, Spiegel A, Nguyen L, Barbazan P, Martin PMV, et al. Cumulative Mortality rates in Aedes polynesiensis after feeding on Polynesian *Wuchereria bancrofti* cCarriers treated with single doses of ivermectin, diethylcarbamazine and placebo. Tropical Medicine and Parasitology. 1991;42(4):343-5.
73. Cartel JL, Spiegel A, Nguyen L, Genelle B, Roux JF. Doubleblindstudy on efficacy and safety of single doses of ivermectin and diethylcarbamazine for treatment of Polynesian *Wuchereria bancrofti* Carriers - results at 6 months. Tropical Medicine and Parasitology. 1991;42(1):38-40.
74. Cartel JL, Spiegel A, Nguyen L, Plichart R, Martin PMV, Roux JF. The treatment of microfilaremia due to *Wuchereria bancrofti* var. *pacifica*: ivermectin versus diethylcarbamazine. Annales de la Société Belge de Médecine Tropicale. 1991;71(3):229-36.
75. Cartel JL, Spiegel A, Nguyen Ngnoc L, Cardines R, Plichart R, Martin PM, et al. Single versus repeated doses of ivermectin and diethylcarbamazine for the treatment of *Wuchereria bancrofti* var. *pacifica* microfilaremia. Results at 12 months of a double-blind study. Tropical Medicine and Parasitology. 1991;42(4):335-8.
76. Cartel JL, Spiegel A, Nguyen Ngnoc L, Cardines R, Plichart R, Martin PM, et al. Compared efficacy of repeated annual and semi-annual doses of ivermectin and diethylcarbamazine for prevention of *Wuchereria bancrofti* filariasis in French Polynesia. Final evaluation. Tropical Medicine and Parasitology. 1992;43(2):91-4.
77. Casley-Smith JR, Casley-Smith JR. Modern treatment of lymphoedema. II. The benzopyrones. Australian Journal of Dermatology. 1992;33(2):69-74.
78. Cattani J, Taufa T, Anderson W, Lourie J. Malaria and filariasis in the Ok Tedi Region of the Star Mountains, Papua New Guinea. Papua New Guinea Medical Journal. 1983;26(2):122-6.
79. Chambers EW, Bossin HC, Ritchie SA, Russell RC, Dobson SL. Landing response of *Aedes (Stegomyia) polynesiensis* mosquitoes to coloured targets. Medical and Veterinary Entomology. 2013;27(3):332-8.
80. Chambers EW, Bossin HC, Ritchie SA, Russell RC, Dobson SL. The impact of insecticide-treated cloth targets on the survival of *Stegomyia polynesiensis* (= *Aedes polynesiensis*) under laboratory and semi-field conditions in French Polynesia. Medical and Veterinary Entomology. 2016;30(3):247-52.
81. Chambers EW, Hapairai L, Peel BA, Bossin H, Dobson SL. Male mating competitiveness of a *Wolbachia*-introgressed *Aedes polynesiensis* strain under semi-field conditions. PLoS Neglected Tropical Diseases. 2011;5(8):e1271.
82. Chambers EW, McClintock SK, Avery MF, King JD, Bradley MH, Schmaedick MA, et al. Xenomonitoring of *Wuchereria bancrofti* and *Dirofilaria immitis* infections in mosquitoes from American Samoa: trapping considerations and a comparison of polymerase chain reaction assays with dissection. American Journal of Tropical Medicine and Hygiene. 2009;80(5):774-81.
83. Chanteau S, Glaziou P, Mouliapelat JP, Plichart C, Luquiaud P, Cartel JL. Low positive predictive value of anti-*Brugia malayi* IgG and IgG4 serology for the diagnosis of *Wuchereria bancrofti*. Transactions of the Royal Society of Tropical Medicine and Hygiene. 1994;88(6):661-2.
84. Chanteau S, Glaziou P, Plichart C, Luquiaud P, Moulia-Pelat JP, N'Guyen L, et al. *Wuchereria bancrofti* filariasis in French Polynesia: age-specific patterns of microfilaremia, circulating antigen, and specific IgG and IgG4 responses according to transmission level. International Journal for Parasitology. 1995;25(1):81-5.
85. Chanteau S, Guidi C, Durosoir JL. Efficiency of papain-treated microfilariae of *Wuchereria bancrofti* (var. *pacifica*) as antigen for serodiagnosis of bancroftian filariasis in French Polynesia. Transactions of the Royal Society of Tropical Medicine and Hygiene. 1986;80(5):795-9.
86. Chanteau S, Mouliapelat JP, Glaziou P, Nguyen NL, Luquiaud P, Plichart C, et al. Og4C3 Circulating antigen - a marker of infection and adult worm burden in *Wuchereria bancrofti* filariasis. Journal of Infectious Diseases. 1994;170(1):247-50.
87. Chanteau S, Plichart R, Spiegel A, Martin PMV, Cartel JL. Diagnostic values of Elisa-IgG4 as compared to Elisa-IgG and indirect immunofluorescence, for the routine diagnosis of bancroftian filariasis in the South Pacific - application on capillary blood collected on filter paper. Tropical Medicine and Parasitology. 1991;42(4):339-42.
88. Chanteau S, Roux JF. [Bancroftian lymphatic filariasis: toward its elimination from the Pacific?]. Bulletin de la Société de Pathologie Exotique et de ses Filiales. 2008;101(3):254-60.
89. Charlwood JD. Survival rate variation of *Anopheles farauti* (Diptera: Culicidae) between neighboring villages in coastal Papua New Guinea. Journal of Medical Entomology. 1986;23(4):361-5.
90. Charlwood JD. A differential response to mosquito nets by *Anopheles* and *Culex* mosquitoes from Papua New Guinea. Transactions of the Royal Society of Tropical Medicine and Hygiene. 1986;80(6):958-60.
91. Charlwood JD, Bryan JH. A mark-recapture experiment with the filariasis vector *Anopheles punctulatus* in Papua New Guinea. Annals of Tropical Medicine and Parasitology. 1987;81(4):429-36.
92. Charlwood JD, Dagoro H. Impregnated bed nets for the control of filariasis transmitted by *Anopheles punctulatus* in rural Papua New Guinea. Papua New Guinea Medical Journal. 1987;30(3):199-202.
93. Charlwood JD, Graves PM. The effect of permethrin-impregnated bednets on a population of *Anopheles farauti* in coastal Papua New Guinea. Medical and Veterinary Entomology. 1987;1(3):319-27.
94. Charlwood JD, Graves PM, Alpers MP. The ecology of the *Anopheles punctulatus* group of mosquitoes from Papua New Guinea: a review of recent work. Papua New Guinea Medical Journal J. 1986;29(1):19-26.
95. Charlwood JD, Graves PM, Marshall TF. Evidence for a 'memorized' home range in *Anopheles farauti* females from Papua New Guinea. Medical and Veterinary Entomology. 1988;2(2):101-8.
96. Charlwood JD, Paru R, Dagoro H, Lagog M. Influence of moonlight and gonotrophic age on biting activity of *Anopheles farauti* (Diptera: Culicidae) from Papua New Guinea. Journal of Medical Entomology. 1986;23(2):132-5.
97. Charters AD. Filariasis in immigrants in Western Australia, including a case of chyluria recurring in seven pregnancies. Medical Journal of Australia. 1970;2(20):919-22.
98. Charters AD. Tropical infections contracted in Papua New Guinea and imported into Western Australia. Papua New Guinea Medical Journal. 1981;24(2):74-9.
99. Charters AD, Cullity GJ, Godfrey RC, Roberts WD, Tozer RL. Tropical pulmonary eosinophilia in immigrants in Western Australia. Medical Journal of Australia. 1972;2(21):1195-8.
100. Chaves LF, Imanishi N, Hoshi T. Population dynamics of *Armigeres subalbatus* (Diptera: Culicidae) across a temperate altitudinal gradient. Bulletin of Entomological Research. 2015;105(5):589-97.
101. Chow CY. Filariasis vectors in the Western Pacific region. Zeitschrift für Tropenmedizin und Parasitologie. 1973;24(4):404-18.
102. Chu BK, Deming M, Biritwum NK, Bougma WR, Dorkenoo AM. Transmission assessment surveys (TAS) to define endpoints for lymphatic filariasis mass drug administration: a multicenter evaluation. PLoS Neglected Tropical Diseases. 2013;7(12).
103. Chu BK, Gass K, Batcho W, 'Ake M, Dorkenoo AM, Adjinacou E, et al. Pilot assessment of soil-transmitted helminthiasis in the context of Transmission Assessment Surveys for lymphatic filariasis in Benin and Tonga. PLoS Neglected Tropical Diseases. 2014;8(2).
104. Cooper RD, Frances SP, Popat S, Waterson DG. The effectiveness of light, 1-octen-3-ol, and carbon dioxide as attractants for anopheline mosquitoes in Madang Province, Papua New Guinea. Journal of the American Mosquito Control Association. 2004;20(3):239-42.
105. Cooper RD, Frances SP, Waterson DGE, Piper RG, Sweeney AW. Distribution of anopheline mosquitoes in northern Australia. Journal of the American Mosquito Control Association. 1996;12(4):656-63.
106. Cooper RD, Waterson DGE, Frances SP, Beebe NW, Pluess B, Sweeney AW. Malaria vectors of Papua New Guinea. International Journal for Parasitology. 2009;39(13):1495-501.
107. Cooper RD, Waterson DGE, Frances SP, Beebe NW, Sweeney AW. The anopheline fauna of Papua New Guinea. Journal of the American Mosquito Control Association. 2006;22(2):213-21.
108. Cooper RD, Waterson DGE, Kupo M, Foley DH, Beebe NW, Sweeney AW. Anopheline mosquitoes of the western province of Papua New Guinea. Journal of the American Mosquito Control Association. 1997;13(1):5-12.
109. Crainey JL, Ribeiro da Silva TR, Luz SL. Historic accounts of *Mansonella* parasitaemias in the South Pacific and their relevance to lymphatic filariasis elimination efforts today. Asian Pacific Journal of Tropical Medicine. 2016;9(3):205-10.
110. Crump A. PacELF - Pioneering disease elimination. Appropriate Technology.. 2005 Sept 2005:3.
111. Cuenco KT, Ottesen EA, Williams SA, Nutman TB, Steel C. Heritable factors play a major role in determining host responses to *Wuchereria bancrofti* infection in an isolated South Pacific island population.Journal of Infectious Diseases. 2009;200(8):1271-8.
112. Daures M, Champagnat J, Pfannstiel A, Ringuenoire F, Grangeon JP, Musso D. Filariasis serosurvey, New Caledonia, South Pacific, 2013. Parasites and Vectors. 2015;8:102.
113. Day KP. The endemic normal in lymphatic filariasis - a static concept. Parasitology Today. 1991;7(12):341-3.
114. Day KP, Gregory WF, Maizels RM. Age-specific acquisition of immunity to infective larvae in a bancroftian filariasis endemic area of Papua New Guinea. Parasite Immunology. 1991;13(3):277-90.
115. Day KP, Grenfell B, Spark R, Kazura JW, Alpers MP. Age specific patterns of change in the dynamics of *Wuchereria bancrofti* infection in Papua Ne Guinea. American Journal of Tropical Medicine and Hygiene. 1991;44(5):518-27.
116. Day KP, Spark R, Garner P, Raiko A, Wenger JD, Weiss N, et al. Serological evaluation of the macrofilaricidal effects of diethylcarbamazine treatment in bancroftian filariasis. American Journal of Tropical Medicine and Hygiene. 1991;44(5):528-35.
117. Dean M. Port Vila - Launching a lymphatic filariasis campaign in the Pacific Islands. Lancet. 2000;356(9224):143.
118. Dean M. Pacific nations lead the way in fighting lymphatic filariasis. Lancet. 2003;362(9399):1906-.
119. Desowitz RS, Berman SJ, Puloka T. Hyperendemic subperiodic bancroftian filariasis: a search for clinical and immunological correlates of microfilaraemia. Bulletin of the World Health Organization. 1976;54(5):565-71.
120. Desowitz RS, Hitchcock JC. Hyperendemic bancroftian filariasis in the Kingdom of Tonga: the application of the membrane filter concentration technique to an age-stratified blood survey. American Journal of Tropical Medicine and Hygiene. 1974;23(5):877-9.
121. Desowitz RS, Jenkins C, Anian G. Bancroftian filariasis in an isolated hunter gatherer shifting horticulturist group in Papua New Guinea. Bulletin of the World Health Organization. 1993;71(1):55-8.
122. Desowitz RS, Southgate BA. Studies of filariasis in the Pacific. 2. The persistence of microfilaraemia in diethylcarbamazine treated populations of Fiji and Western Samoa: diagnostic application of the membrane-filtration technique. Southeast Asian Journal of Tropical Medicine and Public Health. 1973;4(2):179-83.
123. Desowitz RS, Southgate BA, Mataika JU. Studies on filariasis in the Pacific. 3. Comparative efficacy of the stained blood-film, counting-chamber and membrane-filtration techniques for the diagnosis of *Wuchereria bancrofti* microfilaraemia in untreated patients in areas of low endemicity. Southeast Asian Journal of Tropical Medicine and Public Health. 1973;4(3):329-35.
124. Dissanayake S, Forsyth KP, Ismail MM, Mitchell GF. Detection of circulating antigen in bancroftian filariasis by using a monoclonal antibody. American Journal of Tropical Medicine and Hygiene. 1984;33(6):1130-40.
125. Doherty R. Australia's contribution to tropical health: past and present. Medical Journal of Australia. 1993;158(8):552-7.
126. Durrheim DN, Nelesone T, Speare R, Melrose W. Certifying lymphatic filariasis elimination in the Pacific--the need for new tools. Pacific health Dialog. 2003;10(2):149-54.
127. Dutton TJ, Sinkins SP. Filarial susceptibility and effects of *Wolbachia* in *Aedes pseudoscutellaris* mosquitoes. Medical and Veterinary Entomology. 2005;19(1):60-5.
128. Elliott SA. *Aedes albopictus* in the Solomon and Santa Cruz Islands, South Pacific. Transactions of the Royal Society of Tropical Medicine and Hygiene. 1980;74(6):747-8.
129. Enzenauer RJ, Underwood GH, Jr., Ribbing J. Tropical pulmonary eosinophilia. Southern Medical Journal. 1990;83(1):69-72.
130. Erickson SM, Thomsen EK, Keven JB, Vincent N, Koimbu G, Siba PM, et al. Mosquito-parasite interactions can shape filariasis transmission dynamics and impact elimination programs. PLoS Neglected Tropical Diseases. 2013;7(9):e2433.
131. Esterre P, Plichart C, Huin-Blondey MO, Nguyen L. Role of streptococcal infection in the acute pathology of lymphatic filariasis. Parasite - Journal de la Société Française de Parasitologie. 2000;7(2):91-4.
132. Esterre P, Plichart C, Huin-Blondey MO, Nguyen LN, Hartmann D, Guerret S, et al. Circulating fibrosis markers, eosinophil cationic protein and eosinophil protein X in patients with *Wuchereria bancrofti* infection: association with clinical status. Parasite - Journal de la Société Française de Parasitologie. 2006;13(2):165-70.
133. Esterre P, Plichart C, Sechan Y, Nguyen NL. The impact of 34 years of massive DEC chemotherapy on *Wuchereria bancrofti* infection and transmission: the Maupiti cohort. Tropical Medicine and International Health. 2001;6(3):190-5.
134. Esterre P, Vigneron E, Roux J. [The history of lymphatic filarlasis control programme in French Polynesia: lessons from a 50 years effort]. Bulletin de la Société de Eathologie exotique et de ses Filiales. 2005;98(1):41-50.
135. Ewers WH. Parasites of man in Papua New Guinea. Southeast Asian Journal of Tropical Medicine and Public Health. 1972;3(1):79-86.
136. Failloux AB, Raymond M, Ung A, Chevillon C, Pasteur N. Genetic differentiation associated with commercial traffic in the Polynesian mosquito, *Aedes polynesiensis* Marks 1951. Biological Journal of the Linnean Society. 1997;60(1):107-18.
137. Failloux AB, Raymond M, Ung A, Glaziou P, Martin PMV, Pasteur N. Variation in the vector competence of *Aedes polynesiensis* for *Wuchereria bancrofti*. Parasitology. 1995;111:19-29.
138. Failloux AB, Rodhain F. Importance of mosquito population genetic studies in medical entomology. Annales de la Société Entomologique de France. 1999;35(1):1-16.
139. Fauran P, Lacoste J, Combes D, Marcille P, Charpin M. [*Wuchereria bancrofti* human aperiodic filariasis in French territory of Wallis and Futuna (author's transl)]. Médecine Tropicale: Revue du Corps de Santé Colonial. 1981;41(6):665-9.
140. Foley DH, Meek SR, Bryan JH. The *Anopheles punctulatus* group of mosquitoes in the Solomon Islands and Vanuatu surveyed by allozyme electrophoresis. Medical and Veterinary Entomology. 1994;8(4):340-50.
141. Foley DH, Paru R, Dagoro H, Bryan JH. Allozyme analysis reveals six species within the *Anopheles punctulatus* complex of mosquitoes in Papua New Guinea. Medical and Veterinary Entomology. 1993;7(1):37-48.
142. Forsyth K. New approaches to the control of lymphatic filariasis using diethylcarbamazine. Papua New Guinea Medical Journal. 1987;30(3):189-91.
143. Forsyth KP, Spark R, Kazura J, Brown GV, Peters P, Heywood P, et al. A monoclonal antibody-based immunoradiometric assay for detection of circulating antigen in bancroftian filariasis. Journal of Immunology. 1985;134(2):1172-7.
144. Frances SP, Baade LM, Kubofcik J, Nutman TB, Melrose WD, McCarthy JS, et al. Seroconversion to filarial antigens in Australian defence force personnel in Timor Leste. American Journal of Tropical Medicine and Hygiene. 2008;78(4):560-3.
145. Frances SP, Waterson DG, Beebe NW, Cooper RD. Field evaluation of repellent formulations containing DEET and picaridin against mosquitoes in Northern Territory, Australia. Journal of Medical Entomology. 2004;41(3):414-7.
146. Fraser M, Taleo G, Taleo F, Yaviong J, Amos M, Babu M, et al. Evaluation of the program to eliminate lymphatic filariasis in Vanuatu following two years of mass drug administration implementation: results and methodologic approach. American Journal of Tropical Medicine and Hygiene. 2005;73(4):753-8.
147. Fujii Y, Kaneko S, Nzou SM, Mwau M, Njenga SM, Tanigawa C, et al. Serological surveillance development for tropical infectious diseases using simultaneous microsphere-based multiplex assays and finite mixture models. PLoS Neglected Tropical Diseases. 2014;8(7):e3040.
148. Journal of HelminthologyFukumoto H. [Follow-up study on the outcome of filarial mass treatment]. Fukuoka Igaku Zasshi. 1984;75(8):484-95.
149. Gambhir M, Bockarie M, Tisch D, Kazura J, Remais J, Spear R, et al. Geographic and ecologic heterogeneity in elimination thresholds for the major vector-borne helminthic disease, lymphatic filariasis. BMC Biology. 2010;8(22).
150. Gambhir M, Michael E. Complex ecological dynamics and eradicability of the vector borne macroparasitic disease, lymphatic filariasis. PLoS One. 2008;3(8):e2874.
151. Gass K, Beau de Rochars MV, Boakye D, Bradley M, Fischer PU, Gyapong J, et al. A multicenter evaluation of diagnostic tools to define endpoints for programs to eliminate bancroftian filariasis. PLoS Neglected Tropical Diseases. 2012;6(1):e1479.
152. Gasser RB. Bancroft--Mackerras oration. Molecular taxonomic, diagnostic and genetic studies of parasitic helminths. International Journal for Parasitology. 2001;31(9):860-4.
153. Gasser RB, LeGoff L, Petit G, Bain O. Rapid delineation of closely-related filarial parasites using genetic markers in spacer rDNA. Acta Tropica. 1996;62(3):143-50.
154. Glaziou P, Mouliapelat JP, Nguyen LN, Chanteau S, Martin PMV, Cartel JL. Double-blind controlled trial of a single dose of the combination ivermectin 400-Mu-G/Kg plus diethylcarbamazine 6mg/Kg for the treatment of bancroftian filariasis - results at 6 months. Transactions of the Royal Society of Tropical Medicine and Hygiene. 1994;88(6):707-8.
155. Goldsmid JM. Imported disease: a continuing and increasing threat to Australia. Social Science & Medicine: Medical Geography. 1980;14(2):101-9.
156. Goldsmid JM, Nightingale R, Clark D. Imported filarial infections in Tasmania. Medical Journal of Australia. 1980;1(13):667.
157. Gordon S, Melrose W, Warner J, Buttner P, Ward L. Lymphatic filariasis: a method to identify subclinical lower limb change in PNG adolescents. PLoS Neglected Tropical Diseases. 2011;5(7):e1242.
158. Graves PM, Makita L, Susapu M, Brady MA, Melrose W, Capuano C, et al. Lymphatic filariasis in Papua New Guinea: distribution at district level and impact of mass drug administration, 1980 to 2011. Parasites and Vectors. 2013;6.
159. Graves PM, Wood P, Bossin H. Lymphatic filariasis in Oceania. In: Loukas A, editor. Neglected Tropical Diseases of Oceania. Neglected Tropical Diseases. Switzerland: Springer Verlag.; 2016. p. 101-42.
160. Green DF, Yates JA. Microscopic visualization of *Wuchereria* and *Brugia* larval stages in intact cleared mosquitoes. American Journal of Tropical Medicine and Hygiene. 1994;51(4):483-8.
161. Grenfell BT, Das PK, Rajagopalan PK, Bundy DAP. Frequency distribution of lymphatic filariasis microfilariae in human populations - population processes and statistical Eestimation. Parasitology. 1990;101:417-27.
162. Grenfell BT, Michael E. Infection and disease in lymphatic filariasis: an epidemiological approach. Parasitology. 1992;104 Suppl:S81-90.
163. Grove DI. Worms in Australia. Medical Journal of Australia. 1993;159(7):464-6.
164. Guillo JY. [Pathology in the Republic of Vanuatu]. Bulletin de la Société de Pathologie Exotique et de ses Filiales. 1984;77(2):222-6.
165. Hagiwara K, Toyama K, Miyazato H, Nonaka S. A case of acquired lymphangioma due to a suspected old filariasis and a review of literature. Journal of Dermatology. 1994;21(5):358-62.
166. Hagiya H, Otsuka F. Refractory chyluria due to filariasis. Infection. 2015;43(6):785-6.
167. Hagiya H, Terasaka T, Kimura K, Satou A, Asano K, Waseda K, et al. Filarial chyluria as a rare cause of urinary retention. Internal Medicine. 2014;53(17):2001-5.
168. Hamilton RG, Hussain R, Ottesen EA. Immunoradiometric assay for detection of filarial antigens in human serum. Journal of Immunology. 1984;133(4):2237-42.
169. Hamon J. Epidemiology of bancroftian filariasis in Africa, Asia and Oceania. Annales de la Societe Belge de Médecine Tropicale. 1981;61(2):233-55.
170. Hapairai LK, Joseph H, Sang MAC, Melrose W, Ritchie SA, Burkot TR, et al. Field evaluation of selected traps and lures for monitoring the filarial and arbovirus Vector, *Aedes polynesiensis* (Diptera: Culicidae), in French Polynesia. Journal of Medical Entomology. 2013;50(4):731-9.
171. Hapairai LK, Plichart C, Naseri T, Silva U, Tesimale L, Pemita P, et al. Evaluation of traps and lures for mosquito vectors and xenomonitoring of *Wuchereria bancrofti* infection in a high prevalence Samoan village. Parasites and Vectors. 2015;8:287.
172. Hapairai LK, Sang MAC, Bossin HC. Comparison of the Centers for Disease Control and Prevention backpack and Insectazooka aspirators for sampling *Aedes polynesiensis* in French Polynesia. Journal of the American Mosquito Control Association. 2014;30(2):126-9.
173. Hapairai LK, Sang MAC, Sinkins SP, Bossin HC. Population studies of the filarial vector *Aedes polynesiensis* (Diptera: Culicidae) in two island settings of French Polynesia. Journal of Medical Entomology. 2013;50(5):965-76.
174. Harrington H, Asugeni J, Jimuru C, Gwalaa J, Ribeyro E, Bradbury R, et al. A practical strategy for responding to a case of lymphatic filariasis post-elimination in Pacific Islands. Parasites and Vectors. 2013;6:218.
175. Hawking F, Denham DA. The distribution of human filariasis throughout the world. Part I. the Pacific Region, including New Guinea. Tropical Diseases Bulletin. 1976;73(5):347-73.
176. Hawking F, Jennings T, Louis FJ, Tuira E. The mechanisms which affect the periodic cycle of Pacific *Wuchereria bancrofti* microfilariae. Journal of Helminthology. 1981;55(2):95-100.
177. Hayashi S. Brugian filariasis in Japan. Tropical Medicine and Health. 2011;39(1 Suppl 2):25-8.
178. Helmy H, Weil GJ, Faris R, Gad AM, Chandrashekar R, Ashour A, et al. Human antibody responses to *Wuchereria bancrofti* infective larvae. Parasite Immunology. 2000;22(2):89-96.
179. Hemer SR. Health care and illness in Lihir, New Ireland Province, in the context of the development of the Lihir gold mine. Papua New Guinea Medical Journal. 2005;48(3-4):188-95.
180. Henry-Halldin CN, Nadesakumaran K, Keven JB, Zimmerman AM, Siba P, Mueller I, et al. Multiplex assay for species identification and monitoring of insecticide resistance in *Anopheles punctulatus* group populations of Papua New Guinea. American Journal of Tropical Medicine and Hygiene. 2012;86(1):140-51.
181. Henry-Halldin CN, Reimer L, Thomsen E, Koimbu G, Zimmerman A, Keven JB, et al. High throughput multiplex assay for species identification of Papua New Guinea malaria vectors: members of the *Anopheles punctulatus* (Diptera: Culicidae) species group. American Journal of Tropical Medicine and Hygiene. 2011;84(1):166-73.
182. Hii J, Bockarie MJ, Flew S, Genton B, Tali A, Dagoro H, et al. The epidemiology and control of lymphatic filariasis on Lihir Island, New Ireland Province. Papua New Guinea Medical Journal. 2000;43(3-4):188-95.
183. Hii JLK, Kanai L, Foligela A, Kan SKP, Burkot TR, Wirtz RA. Impact of permethrin-impregnated mosquito nets compared with DDT house-spraying against malaria transmission by *Anopheles farauti* and *An. punctulatus* in the Solomon Islands. Medical and Veterinary Entomology. 1993;7(4):333-8.
184. Hii JLK, Smith T, Mai A, Ibam E, Alpers MP. Comparison between anopheline mosquitoes (Diptera : Culicidae) caught using different methods in a malaria endemic area of Papua New Guinea. Bulletin of Entomological Research. 2000;90(3):211-9.
185. Hii JLK, Smith T, Mai A, Mellor S, Lewis D, Alexander N, et al. Spatial and temporal variation in abundance of *Anopheles* (Diptera: Culicidae) in a malaria endemic area in Papua New Guinea. Journal of Medical Entomology. 1997;34(2):193-205.
186. Hirshman JH. Communicable disease in the South Pacific Islands, 1. Medical Journal of Australia. 1976;2(20):758-60.
187. Hirshman JH. Communicable disease in the South Pacific islands, 2. Medical Journal of Australia. 1976;2(21):794-8.
188. Hise AG, Gillette-Ferguson I, Pearlman E. The role of endosymbiotic *Wolbachia* bacteria in filarial disease. Cellular Microbiology. 2004;6(2):97-104.
189. Hise AG, Hazlett FE, Bockarie MJ, Zimmerman PA, Tisch DJ, Kazura JW. Polymorphisms of innate immunity genes and susceptibility to lymphatic filariasis. Genes and Immunity. 2003;4(7):524-7.
190. Hitchcock JC. Transmission of sub-periodic filariasis in Tonga by *Aedes oceanicus* Belkin. Transactions of the Royal Society of Tropical Medicine and Hygiene. 1971;65(3):408-9.
191. Hornabrook RW, Kelly A, McMillan B. Parasitic infection of man on Kar Kar Island, New Guinea. American Journal of Tropical Medicine and Hygiene. 1975;24(4):590-5.
192. Horton J, Witt C, Ottesen EA, Lazdins JK, Addiss DG, Awadzi K, et al. An analysis of the safety of the single dose, two drug regimens used in programmes to eliminate lymphatic filariasis. Parasitology. 2000;121 Suppl:S147-60.
193. Hotez PJ, Ehrenberg JP. Escalating the global fight against neglected tropical diseases through interventions in the Asia Pacific region. Advances in Parasitology. 2010;72:31-53.
194. Houston R. Salt fortified with diethylcarbamazine (DEC) as an effective intervention for lymphatic filariasis, with lessons learned from salt iodization programmes. Parasitology. 2000;121 Suppl:S161-73.
195. Hughes RG, Sharp DS, Hughes MC, Akau'ola S, Heinsbroek P, Velayudhan R, et al. Environmental influences on helminthiasis and nutritional status among Pacific schoolchildren. International Journal of Environmental Health Research. 2004;14(3):163-77.
196. Huppatz C, Capuano C, Palmer K, Kelly PM, Durrheim DN. Lessons from the Pacific Programme to Eliminate Lymphatic Filariasis: a case study of 5 countries. BMC Infectious Diseases. 2009;9:92.
197. Huppatz C, Durrheim D, Lammie P, Kelly P, Melrose W. Eliminating lymphatic filariasis - the surveillance challenge. Tropical Medicine and International Health. 2008;13(3):292-4.
198. Ichimori K. Entomology of the filariasis control programme in Samoa, *Aedes polynesiensis* and *Ae. samoanus*. Medical Entomology and Zoology. 2001;52(1):11-21.
199. Ichimori K. MDA - Lymphatic Filariasis. Tropical Medicine and Health. 2014;42(2 Suppl):21-4.
200. Ichimori K, Crump A. Pacific collaboration to eliminate lymphatic filariasis. Trends in Parasitology. 2005;21(10):441-4.
201. Ichimori K, Graves PM, Crump A. Lymphatic filariasis elimination in the Pacific: PacELF replicating Japanese success. Trends in Parasitology. 2007;23(1):36-40.
202. Ichimori K, King JD, Engels D, Yajima A, Mikhailov A, Lammie P, et al. Global Programme to Eliminate Lymphatic Filariasis: the processes underlying programme success. PLoS Neglected Tropical Diseases. 2014;8(12).
203. Ichimori K, Tupuimalagi-Toelupe P, Iosia VT, Graves PM. *Wuchereria bancrofti* filariasis control in Samoa before PacELF (Pacific Programme to Eliminate Lymphatic Filariasis). Tropical Medicine and Health. 2007;35(3):261-9.
204. Ito S, Natori C, Nagata A, Ohara A. [Case report; A case of filarial chyluria with severe hypoproteinemia]. Nihon Naika Gakkai Zasshi. 2013;102(12):3227-9.
205. Itoh M, Weerasooriya MV, Qiu G, Gunawardena NK, Anantaphruti MT, Tesana S, et al. Sensitive and specific enzyme-linked immunosorbent assay for the diagnosis of *Wuchereria bancrofti* infection in urine samples. American Journal of Tropical Medicine and Hygiene. 2001;65(4):362-5.
206. Jacquemart Y, Josse R. [Papua New Guinea]. Médecine Tropicale: Revue du Corps de Santé Colonial. 2002;62(6):583-8.
207. Jeannin C, Paoaafaite T, Frogier H, Tetuanui A, Faaruia M, Marie J, et al. Assessing the efficacy of deltamethrin-impregnated lethal targets for the control of the lymphatic filariasis vector in Tahiti, French Polynesia. American Journal of Tropical Medicine and Hygiene. 2010;83(5):299-.
208. Jenkins CL. Health in the early contact period: a contemporary example from Papua New Guinea. Social Science and Medicine. 1988;26(10):997-1006.
209. Jeremiah CJ, Aboltins CA, Stanley PA. Lymphatic filariasis in Australia: an update on presentation, diagnosis and treatment. Medical Journal of Australia. 2011;194(12):655-7.
210. Joseph H, Clough A, Peteru A, Crawley S, Pulu T, Maiava F, et al. Exploratory study investigating factors influencing mass drug administration (MDA) compliance for lymphatic filariasis in Samoa. Samoa Medical Journal. 2010;2(3):12-25.
211. Joseph H, Maiava F, Naseri T, Silva U, Lammie P, Melrose W. Epidemiological assessment of continuing transmission of lymphatic filariasis in Samoa. Annals of Tropical Medicine and Parasitology. 2011;105(8):567-78.
212. Joseph H, Maiava F, Naseri T, Taleo F, ake M, Capuano C, et al. Application of the filariasis CELISA antifilarial IgG(4) antibody assay in surveillance in lymphatic filariasis elimination programmes in the South Pacific. Journal of Tropical Medicine. 2011;2011:492023.
213. Joseph H, Melrose WD. Applicability of the filter paper technique for detection of antifilarial IgG4 antibodies using the Bm14 Filariasis CELISA. Journal of Parasitology Research. 2010;2010.
214. Joseph H, Moloney J, Maiava F, McClintock S, Lammie P, Melrose W. First evidence of spatial clustering of lymphatic filariasis in an Aedes polynesiensis endemic area. Acta Tropica. 2011;120 Suppl 1:S39-47.
215. Joseph HM, Melrose W. Applicability of the filter paper technique for detection of antifilarial IgG(4) antibodies using the Bm14 filariasis CELISA. Journal of Parasitology Research. 2010;2010.
216. Kaeuffer H, Carme B, Laigret J. [Significance of passive hemagglutination and its prospectives in lymphatic filariasis]. Bulletin de la Société de Pathologie Exotique et de ses Filiales. 1976;69(3):244-57.
217. Kazura J, Greenberg J, Perry R, Weil G, Day K, Alpers M. Comparison of single-dose diethylcarbamazine and ivermectin for treatment of bancroftian filariasis in Papua New Guinea. American Journal of Tropical Medicine and Hygiene. 1993;49(6):804-11.
218. Kazura JW. Filariasis and onchocerciasis. Current Opinion in Infectious Diseases. 1997;10(5):341-4.
219. Kazura JW. Lymphatic filariasis in Papua New Guinea. Papua New Guinea Medical Journal. 2000;43(3-4):159-60.
220. Kazura JW, Bockarie M, Alexander N, Perry R, Bockarie F, Dagoro H, et al. Transmission intensity and its relationship to infection and disease due to *Wuchereria bancrofti* in Papua New Guinea. Journal of Infectious Diseases. 1997;176(1):242-6.
221. Kazura JW, Bockarie MJ. Lymphatic filariasis in Papua New Guinea: interdisciplinary research on a national health problem. Trends in Parasitology. 2003;19(6):260-3.
222. Kazura JW, Hazlett FE, Jr., Pearlman E, Day K, el-Zeiny A, Nilsen TW, et al. Antigenicity of a protective recombinant filarial protein in human bancroftian filariasis. Journal of Infectious Diseases. 1992;166(6):1453-7.
223. Kazura JW, Spark R, Forsyth K, Brown G, Heywood P, Peters P, et al. Parasitologic and clinical features of bancroftian filariasis in a community in East Sepik Province, Papua New Guinea. American Journal of Tropical Medicine and Hygiene. 1984;33(6):1119-23.
224. Keating J, Yukich JO, Mollenkopf S, Tediosi F. Lymphatic filariasis and onchocerciasis prevention, treatment, and control costs across diverse settings: A systematic review. Acta Tropica. 2014;135:86-95.
225. Kessel JF. A review of the filariasis control programme in Tahiti from November 1967 to January 1968. Bulletin of the World Health Organization. 1971;44(6):783-94.
226. Kessel JF, Siliga N, Tompkins H, Jr., Jones K. Periodic mass treatment with diethylcarbamazine for the control of filariasis in American Samoa. Bulletin of the World Health Organization. 1970;43(6):817-25.
227. Keven JB, Reimer L, Katusele M, Koimbu G, Vinit R, Vincent N, et al. Plasticity of host selection by malaria vectors of Papua New Guinea. Parasites and Vectors. 2017;10(1):95.
228. Kimura E. The Global Programme to Eliminate Lymphatic Filariasis: History and achievements with special reference to annual single-dose treatment with diethylcarbamazine in Samoa and Fiji. Tropical Medicine and Health. 2011;39(1):17-30.
229. Kimura E, Itoh M. Filariasis in Japan some 25 years after its eradication. Tropical Medicine and Health. 2011;39(1 Suppl 2):57-63.
230. Kimura E, Mataika JU. Control of lymphatic filariasis by annual single-dose diethylcarbamazine treatments. Parasitology Today. 1996;12(6):240-4.
231. Kimura E, Penaia L, Samarawickrema WA, Spears GFS. Low-density microfilaraemia in subperiodic bancroftian filariasis in Samoa. Bulletin of the World Health Organization. 1985;63(6):1089-96.
232. Kimura E, Penaia L, Spears GF. Comparison of methods for the detection of microfilariae of *Wuchereria bancrofti* in Western Samoa. The Southeast Asian Journal of Tropical Medicine and Public Health. 1984;15(2):167-74.
233. Kimura E, Penaia L, Spears GF. The efficacy of annual single-dose treatment with diethylcarbamazine citrate against diurnally subperiodic bancroftian filariasis in Samoa. Bulletin of the World Health Organization. 1985;63(6):1097-106.
234. Kimura E, Penaia L, Spears GFS. Epidemiology of subperiodic bancroftian filariasis in Samoa 8 years after control by mass treatment with diethylcarbamazine. Bulletin of the World Health Organization. 1985;63(5):869-80.
235. Kimura E, Remit K, Fujiwara M, Aniol K, Siren N. Parasitological and clinical Studies on *Wuchereria bancrofti* infection in Chuuk (Formerly Truk) State, Federated States of Micronesia. Tropical Medicine and Parasitology. 1994;45(4):344-6.
236. Kimura E, Spears GFS, Singh KI, Samarawickrema WA, Penaia L, Sone PF, et al. Long-term efficacy of single-dose mass treatment with diethylcarbamazine citrate against diurnally subperiodic *Wuchereria bancrofti*: eight years' experience in Samoa. Bulletin of the World Health Organization. 1992;70(6):769-76.
237. King CL. Human immune responses to lymphatic filariasis in Papua New Guinea. Papua New Guinea Medical Journal. 2000;43(3-4):203-12.
238. King CL. Transmission intensity and human immune responses to lymphatic filariasis. Parasite Immunology. 2001;23(7):363-71.
239. King CL, Connelly M, Alpers MP, Bockarie M, Kazura JW. Transmission intensity determines lymphocyte responsiveness and cytokine bias in human lymphatic filariasis. Journal of Immunology. 2001;166(12):7427-36.
240. King CL, Nutman TB. Regulation of the immune response in lymphatic filariasis and onchocerciasis. Immunoparasitology Today. 1991(3):A54-A8.
241. King JD, Zielinski-Gutierrez E, Pa'au M, Lammie P. Improving community participation to eliminate lymphatic filariasis in American Samoa. Acta Tropica. 2011;120 Suppl 1:S48-54.
242. Kinnamon KE, Engle RR, Poon BT, Mccall JW, Dzimianski MT. A new class of anti-filariasis compounds - a preliminary look. Military Medicine. 1994;159(5):368-72.
243. Kita K. [Current trend of drug development for Neglected Tropical Diseases (NTDs)]. Yakugaku Zasshi. 2016;136(2):205-11.
244. Kline K, McCarthy JS, Pearson M, Loukas A, Hotez PJ. Neglected tropical diseases of Oceania: review of their prevalence, distribution, and opportunities for control. PLoS Neglected Tropical Diseases. 2013;7(1):e1755.
245. Kloos ZA, Mehlotra RK, Gray LR, Zikursh MJB, Tisch DJ, Siba P, et al. Multiplex PCR-based evaluation of *Plasmodium* spp. And *Wuchereria bancrofti* infections in Papua New Guinea. American Journal of Tropical Medicine and Hygiene. 2010;83(5):68-.
246. Knight R, Mcadam KPWJ, Matola YG, Kirkham V. Bancroftian filariasis and other parasitic infections in the Middle Fly River region of western Papua New Guinea .1. clinical, parasitological and serological studies. Annals of Tropical Medicine and Parasitology. 1979;73(6):563-76.
247. Kobayashi M, Niimura M, Kanazawa T, Husky MK, Malagueno E, Santana JV. Detection of microfilarial antigen in circulating immune complex from sera of *Wuchereria bancrofti*-infected individuals. American Journal of Tropical Medicine and Hygiene. 1997;57(2):200-4.
248. Koga S, Arakaki Y, Matsuoka M, Ohyama C. Remission of filarial chyluria after treatment of coexistent conditions. Lymphology. 1990;23(3):164-6.
249. Koga S, Nagata Y, Arakaki Y, Matsuoka M, Ohyama C. Lymphangiography of filarial chyluria: injections via right and left feet at different times. British Journal of Urology. 1992;69(3):318.
250. Koga S, Nagata Y, Arakaki Y, Matsuoka M, Ohyama C. Unilateral pedal lymphography in patients with filarial chyluria. BJU International. 2000;85(3):222-3.
251. Krentel A, Fischer PU, Weil GJ. A Review of factors that influence individual compliance with mass drug administration for elimination of lymphatic filariasis. PLoS Neglected Tropical Diseases. 2013;7(11).
252. Kwan-Lim GE, Forsyth KP, Maizels RM. Filarial-specific IgG4 response correlates with active *Wuchereria bancrofti* infection. Journal of Immunology. 1990;145(12):4298-305.
253. Kyelem D, Biswas G, Bockarie MJ, Bradley MH, El-Setouhy M, Fischer PU, et al. Determinants of success in national programs to eliminate lymphatic filariasis: a perspective identifying essential elements and research needs. American Journal of Tropical Medicine and Hygiene. 2008;79(4):480-4.
254. Lagraulet J. [Prophylaxis and treatment of lymphatic filariasis in French Polynesia]. Bulletin de la Société de Pathologie Exotique et de ses Filiales. 1973;66(2):311-20.
255. Lagraulet J. [Survey of elephantiasis on the island of Tahaa (Lee-Side Islands, French Polynesia)]. Bulletin de la Société de Pathologie Exotique et de ses Filiales. 1974;67(1):73-7.
256. Lagraulet J, Bonnin P. [Lymphatic filariasis at Mellicolo (New Hebrides; preliminary survey)]. Bulletin de la Société de Pathologie Exotique et de ses Filiales. 1971;64(2):229-31.
257. Lagraulet J, Outin-Fabre D, Tomasini J. [Study on the possibilities of spreading of lymphatic filariasis in New Caledonia]. Bulletin de la Société de Pathologie Exotique et de ses Filiales. 1971;64(1):95-100.
258. Lambdin B, Schmaedick MA, Burkot TR. Utilization of domestic and natural containers by *Aedes oceanicus* in American Samoa. Journal of Medical Entomology. 2008;45(4):758-62.
259. Lambdin BH, Schmaedick MA, McClintock S, Roberts J, Gurr NE, Marcos K, et al. Dry season production of filariasis and dengue vectors in American Samoa and comparison with wet season production. American Journal of Tropical Medicine and Hygiene. 2009;81(6):1013-9.
260. Laney SJ, Susapu M, Weil GJ, Williams SA, Bockarie MJ. Impact of mass drug administration on *Wuchereria bancrofti* infection rates in mosquitoes in Papua New Guinea. American Journal of Tropical Medicine and Hygiene. 2005;73(6):175-.
261. Langy S, Plichart C, Luquiaud P, Williams SA, Nicolas L. The immunodominant *Brugia malayi* paramyosin as a marker of current infection with *Wuchereria bancrofti* adult worms. Infection and Immunity. 1998;66(6):2854-8.
262. Lanham A, Mwanri M. The curse of lymphatic filariasis: would the continual use of diethylcarbamazine eliminate this scourge in Papua New Guinea? American Journal of Infectious Diseases and Microbiology. 2010;1(1):5-12.
263. Lardeux F, Cheffort J. Behavior of *Wuchereria bancrofti* (Filariidea: Onchocercidae) infective larvae in the vector Aedes polynesiensis (Diptera:Culicidae) in relation to parasite transmission. Journal of Medical Entomology. 1996;33(4):516-24.
264. Lardeux F, Cheffort J. Temperature thresholds and statistical modelling of larval *Wuchereria bancrofti* (Filariidea: Onchocercidae) developmental rates. Parasitology. 1997;114:123-34.
265. Lardeux F, Cheffort J. Ambient temperature effects on the extrinsic incubation period of *Wuchereria bancrofti* in *Aedes polynesiensis*: implications for filariasis transmission dynamics and distribution in French Polynesia. Medical and Veterinary Entomology. 2001;15(2):167-76.
266. Lardeux F, Cheffort J. Age-grading and growth of *Wuchereria bancrofti* (Filariidea: Onchocercidae) larvae by growth measurements and its use for estimating blood-meal intervals of its Polynesian vector *Aedes polynesiensis* (Diptera: Culicidae). International Journal for Parasitology. 2002;32(6):705-16.
267. Lardeux F, Nguyen NL, Cartel JL. *Wuchereria bancrofti* (Filariidea, Dipetalonematidae) and its vector Aedes polynesiensis (Diptera, Culicidae) in a French Polynesian village. Journal of Medical Entomology. 1995;32(3):346-52.
268. Lardeux F, Riviere F, Sechan Y, Loncke S. Control of the *Aedes* vectors of the dengue viruses and *Wuchereria bancrofti*: the French Polynesian experience. Annals of Tropical Medicine and Parasitology. 2002;96 Suppl 2:S105-16.
269. Lau CL, Won KY, Becker L, Soares Magalhaes RJ, Fuimaono S, Melrose W, et al. Seroprevalence and spatial epidemiology of lymphatic filariasis in American Samoa after successful mass drug administration. PLoS Neglected Tropical Diseases. 2014;8(11):e3297.
270. Lau CL, Won KY, Lammie PJ, Graves PM. Lymphatic Filariasis Elimination in American Samoa: evaluation of molecular xenomonitoring as a surveillance tool in the endgame. PLoS Neglected Tropical Diseases. 2016;10(11):e0005108.
271. Lau M, Tauchi P, Kim M, Liu F, Namiki T. Filariasis of the breast in a pregnant woman diagnosed by fine-needle aspiration cytology: a case report. Infectious Diseases in Obstetrics and Gynaecology. 1995;3(6):245-7.
272. Le Godinec G, Fauran P. [Survey on filariasis in New Caledonia]. Bulletin de la Société de Pathologie Exotique et de ses Filiales. 1984;77(3):344-51.
273. Leggat PA, Melrose W. Lymphatic filariasis: disease outbreaks in military deployments from World War II. Military Medicine. 2005;170(7):585-9.
274. Leggat PA, Melrose W, Durrheim DN. Could it be lymphatic filariasis? Journal of Travel Medicine. 2004;11(1):56-60.
275. Liang JL, King JD, Ichimori K, Handzel T, Pa'au M, Lammie PJ. Impact of five annual rounds of mass drug administration with diethylcarbamazine and albendazole on *Wuchereria bancrofti* infection in American Samoa. American Journal of Tropical Medicine and Hygiene. 2008;78(6):924-8.
276. Lim KH, Speare R, Thomas G, Graves P. Surgical treatment of genital manifestations of lymphatic filariasis: a systematic review. World Journal of Surgery. 2015;39(12):2885-99.
277. Lim PL, Han P, Chen LH, MacDonald S, Pandey P, Hale D, et al. Expatriates ill after travel: results from the Geosentinel Surveillance Network. BMC Infectious Diseases. 2012;12.
278. Louis FJ, Laigret J. [Trial of the combination of diethylcarbamazine (Notezine)-levamisole (Solaskil) in the control of filariasis by *Wuchereria bancrofti* var. *pacifica* in French Polynesia]. Bulletin de la Société de Pathologie Exotique et de ses Filiales. 1979;72(5-6):471-81.
279. Lunde MN, Paranjape R, Lawley TJ, Ottesen EA. Filarial antigen in circulating immune complexes from patients with *Wuchereria bancrofti* filariasis. American Journal of Tropical Medicine and Hygiene. 1988;38(2):366-71.
280. Maffi M. Contribution to the knowledge of the mosquito fauna of the New Hebrides island group proper (Diptera: Culicidae). Rivista di Parassitologia. 1977;38(2/3):193-214.
281. Maffi M. The mosquitoes (Diptera, Culicidae) of Vanuatu. A further contribution. Rivista di Parassitologia. 1989;6.
282. Mahanty S, Day KP, Alpers MP, Kazura JW. Antifilarial IgG4 antibodies in children from filaria-endemic areas correlate with duration of infection and are dissociated from antifilarial IgE antibodies. Journal of Infectious Diseases. 1994;170(5):1339-43.
283. Mahoney LE, Jr., Aiu P. Filariasis in Samoan immigrants to the United States. American Journal of Tropical Medicine and Hygiene. 1970;19(4):629-31.
284. Mahoney LE, Kessel JF. Treatment failure in filariasis mass treatment programmes. Bulletin of the World Health Organization. 1971;45(1):35-42.
285. Maki J. A review of the long-term in vitro maintenance of adult filarial worms releasing microfilariae. Kitasato Archives of Experimental Medicine. 1991;64(4):179-82.
286. Manguin S, Bangs MJ, Pothikasikorn J, Chareonviriyaphap T. Review on global co-transmission of human *Plasmodium* species and *Wuchereria bancrofti* by *Anopheles* mosquitoes. Infection, Genetics and Evolution. 2010;10(2):159-77.
287. Mataika JU, Dando BC, Spears GFS, Macnamar FN. Mosquito-borne infections in Fiji. 3. Filariasis in Northern Fiji - epidemiological evidence regarding mechanisms of pathogenesis. Journal of Hygiene. 1971;69(2):297-.
288. Mataika JU, Dando BC, Spears GFS, Macnamar FN. Mosquito-borne infections in Fiji .1. Filariasis in Northern Fiji - epidemiological evidence regarding factors influencing prevalence of microfilaraemia of *Wuchereria bancrofti* infections. Journal of Hygiene. 1971;69(2):273-.
289. Mataika JU, Kimura E, Koroivueta J, Shimada M. Efficacy of five annual single doses of diethylcarbamazine for treatment of lymphatic filariasis in Fiji. Bulletin of the World Health Organization. 1998;76(6):575-9.
290. McCarthy JS, Guinea A, Weil GJ, Ottesen EA. Clearance of circulating filarial antigen as a measure of the macrofilaricidal activity of diethylcarbamazine in *Wuchereria bancrofti* infection. Journal of Infectious Diseases. 1995;172(2):521-6.
291. McGreevy PB, Bryan JH, Oothuman P, Kolstrup N. The lethal effects of the cibarial and pharyngeal armatures of mosquitoes on microfilariae. Transactions of the Royal Society of Tropical Medicine and Hygiene. 1978;72(4):361-8.
292. McGuinness B, Soh MC, Kumar S, Donald J. Bag of worms. Australian Radiology. 2007;51 Suppl:B242-5.
293. Mehlotra RK, Gray LR, Blood-Zikursh MJ, Kloos Z, Henry-Halldin CN, Tisch DJ, et al. Short Report: Molecular-based assay for simultaneous detection of four *Plasmodium* spp. and *Wuchereria bancrofti* infections. American Journal of Tropical Medicine and Hygiene. 2010;82(6):1030-3.
294. Melrose W. Lymphatic filariasis: a review 1862-2002. Killarney, Qld: Warwick Educational Publishing Inc, Secretariat, 29 Kurrajong St., Killarney Qld 4373, Australia; 2004. 80 p.
295. Melrose W, Pisters P, Turner P, Kombati Z, Selve BP, Hii J, et al. Prevalence of filarial antigenaemia in Papua New Guinea: results of surveys by the School of Public Health and Tropical Medicine, James Cook University, Townsville, Australia. Papua New Guinea Medical Journal. 2000;43(3-4):161-5.
296. Melrose W, Rahmah N. Use of Brugia Rapid dipstick and ICT test to map distribution of lymphatic filariasis in the Democratic Republic of Timor Leste. Southeast Asian Journal of Tropical Medicine and Public Health. 2006;37(1):22-5.
297. Melrose W, Usurup J, Selve B, Pisters P, Turner P. Development of anti-filarial antibodies in a group of expatriate mine-site workers with varying exposure to the disease. Transactions of the Royal Society of Tropical Medicine and Hygiene. 2000;94(6):706-7.
298. Melrose WD. Lymphatic filariasis: new insights into an old disease. International Journal for Parasitology. 2002;32(8):947-60.
299. Melrose WD. Chemotherapy for lymphatic filariasis: progress but not perfection. Expert Review of Anti-Infective Therapy. 2003;1(4):571-7.
300. Melrose WD, Durrheim DD, Burgess GW. Update on immunological tests for lymphatic filariasis. Trends in Parasitology. 2004;20(6):255-7.
301. Melrose WD, Turner PF, Pisters P, Turner B. An improved Knott's concentration test for the detection of microfilariae. Transactions of the Royal Society of Tropical Medicine and Hygiene. 2000;94(2):176.
302. Mercer DR, Bossin H, Sang MC, O'Connor L, Dobson SL. Monitoring temporal abundance and spatial distribution of *Aedes polynesiensis* using BG-Sentinel traps in neighboring habitats on Raiatea, Society Archipelago, French Polynesia. Journal of Medical Entomology. 2012;49(1):51-60.
303. Mercer DR, Marie J, Bossin H, Faaruia M, Tetuanui A, Sang MC, et al. Estimation of population size and dispersal of *Aedes polynesiensis* on Toamaro Motu, French Polynesia. Journal of Medical Entomology. 2012;49(5):971-80.
304. Mercer DR, Nicolas L, Thiery I. Evaluation of entomopathogenic bacteria against *Aedes polynesiensis*, the vector of lymphatic filariasis in French Polynesia. Journal of the American Mosquito Control Association. 1995;11(4):485-8.
305. Mercer DR, Wettach GR, Smith JL. Effects of larval density and predation by *Toxorhynchites amboinensis* on *Aedes polynesiensis* (Diptera : Culicidae) developing in coconuts. Journal of the American Mosquito Control Association. 2005;21(4):425-31.
306. Michael E, Bundy DA. Global mapping of lymphatic filariasis. Parasitology Today. 1997;13(12):472-6.
307. Michael E, Bundy DA, Grenfell BT. Re-assessing the global prevalence and distribution of lymphatic filariasis. Parasitology. 1996;112 ( Pt 4):409-28.
308. Michael E, Grenfell BT, Bundy DA. The association between microfilaraemia and disease in lymphatic filariasis. Proceedings of the Royal Society of London B: Biological Sciences. 1994;256(1345):33-40.
309. Michael E, Malecela MN, Zervos M, Kazura JW. Global rradication of lymphatic filariasis: the value of chronic disease control in parasite elimination programmes. PLoS One. 2008;3(8).
310. Michael E, Singh BK. Heterogeneous dynamics, robustness/fragility trade-offs, and the eradication of the macroparasitic disease, lymphatic filariasis. BMC Medicine. 2016;14:14.
311. Mitja O, Paru R, Hays R, Griffin L, Laban N, Samson M, et al. The impact of a filariasis control program on Lihir Island, Papua New Guinea. PLoS Neglected Tropical Diseases. 2011;5(8):e1286.
312. Mladonicky JM, King JD, Liang JL, Chambers E, Pa'au M, Schmaedick MA, et al. Assessing transmission of lymphatic filariasis using parasitologic, serologic, and entomologic tools after mass drug administration in American Samoa. American Journal of Tropical Medicine and Hygiene. 2009;80(5):769-73.
313. Moala-Silatolu A, Nakamura K, Seino K, Kizuki M. Greater adherence to mass drug administration against lymphatic filariasis through traditional village forums in Fiji. Journal of Rural Medicine. 2012;7(2):65-72.
314. Monchy D, Barny S, Rougier Y, Baudet JM, Gentile B. [Survey of lymphatic filariasis on Ouvea Island in New Caledonia]. Médecine Tropicale: Revue du Corps de Santé Colonial. 1999;59(2):146-50.
315. Monchy D, Noellat P, Nomoredjo A, Videault A, Dubourdieu D. Nodular breast filariasis: diagnosis by fine needle aspiration. Pathology International. 1996;46(3):228-30.
316. Montresor A, Cong DT, Sinuon M, Tsuyuoka R, Chanthavisouk C, Strandgaard H, et al. Large-scale preventive chemotherapy for the control of helminth infection in Western Pacific countries: six years later. PLoS Neglected Tropical Diseases. 2008;2(8):e278.
317. More SJ, Copeman DB. A highly specific and sensitive monoclonal antibody-based ELISA for the detection of circulating antigen in bancroftian filariasis. Tropical Medicine and Parasitology. 1990;41(4):403-6.
318. More SJ, Copeman DB. Antigen detection ELISAs: pretreatment of serum to reduce interference by specific host antibodies. Tropical Medicine and Parasitology. 1991;42(2):91-4.
319. Moulia-Pelat JP, Glaziou P, Chanteau S, Nguyen-Ngoc L, Marcet Y, Gardines R, et al. Periodicity of *Wuchereria bancrofti* var. *pacifica* filariasis in French Polynesia. Tropical Medicine and Parasitology. 1993;44(2):83-5.
320. Moulia-Pelat JP, Glaziou P, Nguyenngoc L, Cardines D, Spiegel A, Cartel JL. A comparative study of detection methods for evaluation of microfilaremia in lymphatic filariasis control programs. Tropical Medicine and Parasitology. 1992;43(3):146-8.
321. Moulia-Pelat JP, Glaziou P, Weil GJ, Nguyen LN, Gaxotte P, Nicolas L. Combination ivermectin plus diethylcarbamazine, a new effective tool for control of lymphatic filariasis. Tropical Medicine and Parasitology. 1995;46(1):9-12.
322. Moulia-Pelat JP, Nguyen LN, Hascoet H, Luquiaud P, Nicolas L. Advantages of an annual single dose of ivermectin 400 ug/kg plus diethylcarbamazine for community treatment of bancroftian filariasis. Transactions of the Royal Society of Tropical Medicine and Hygiene. 1995;89:682-5.
323. Moulia-Pelat JP, Nguyen LN, Hascoet H, Nicolas L. Combination of ivermectin and diethylcarbamazine for a better control of lymphatic filariasis. Parasite - Journal de la Société Française de Parasitologie. 1996;3(1):45-8.
324. Mount P, Thong M. Perirenal lymphatic filariasis presenting as chyluria during pregnancy. Kidney International. 2006;69(12):2115.
325. Muguruma K, Matsuda T, Koyama Y, Komatz Y. [Chyluria treated with inguinal lymphangiovenous and lymph node-venous anastomosis: a case report]. Nihon Hinyokika Gakkai Zasshi. 1994;85(10):1571-4.
326. Musso D. Relevance of the eosinophil blood count in bancroftian filariasis as a screening tool for the treatment. Pathogens and Global Health. 2013;107(2):96-102.
327. Musso D, Vialette V. Predictive value of the eosinophil counts in the biological diagnosis of lymphatic filariasis in French Polynesia. Médecine et Maladies Infectieuses. 2012;42(12):585-90.
328. Nagaoka F, Itoh M, Samad MS, Takagi H, Weerasooriya MV, Yahathugoda TC, et al. Visual detection of filaria-specific IgG4 in urine using red-colored high density latex beads. Parasitology International. 2013;62(1):32-5.
329. Nakagawa J, Ehrenberg JP, Nealon J, Furst T, Aratchige P, Gonzales G, et al. Towards effective prevention and control of helminth neglected tropical diseases in the Western Pacific Region through multi-disease and multi-sectoral interventions. Acta Tropica. 2015;141:407-18.
330. Nelesone T, Durrheim DN, Speare R, Kiedrzynski T, Melrose WD. Short communication: Strengthening sub-national communicable disease surveillance in a remote Pacific Island country by adapting a successful African outbreak surveillance model. Tropical Medicine and International Health : TM & IH. 2006;11(1):17-21.
331. Nelson GS. Issues in filariasis--a century of enquiry and a century of failure. Acta Tropica. 1981;38(3):197-204.
332. Nguyen NL, Moulia-Pelat JP, Cartel JL. Control of bancroftian filariasis in an endemic area of Polynesia by ivermectin 400 micrograms/kg. Transactions of the Royal Society of Tropical Medicine and Hygiene. 1996;90(6):689-91.
333. Nguyen NL, Mouliapelat JP, Glaziou P, Martin PMV, Cartel JL. Advantages of ivermectin at a single-dose of 400 ug/kg compared with 100 ug/kg for community treatment of ymphatic filariasis in Polynesia. Transactions of the Royal Society of Tropical Medicine and Hygiene. 1994;88(4):461-4.
334. Nguyen NL, Plichart C, Esterre P. Assessment of immunochromatographic test for rapid lymphatic filariasis diagnosis. Parasite. 1999;6(4):355-8.
335. Nicolas L. Bancroftian filariasis: new understanding and strategies for control. Bulletin de l’Institut Pasteur. 1997;95(4):237-46.
336. Nicolas L. New tools for diagnosis and monitoring of bancroftian filariasis parasitism: the Polynesian experience. Parasitology Today. 1997;13(10):370-5.
337. Nicolas L, Langy S, Plichart C, Deparis X. Filarial antibody responses in *Wuchereria bancrofti* transmission area are related to parasitological but not clinical status. Parasite Immunology. 1999;21(2):73-80.
338. Nicolas L, Plichart C, Nguyen LN, Moulia-Pelat JP. Reduction of *Wuchereria bancrofti* adult worm circulating antigen after annual treatments of diethylcarbamazine combined with ivermectin in French Polynesia. Journal of Infectious Diseases. 1997;175(2):489-92.
339. Nicolas L, Scoles GA. Multiplex polymerase chain reaction for detection of *Dirofilaria immitis* (Filariidea: Onchocercidae) and *Wuchereria bancrofti* (Filarioidea: Dipetalonematidae) in their common vector *Aedes polynesiensis* (Diptera: Culicidae). Journal of Medical Entomology. 1997;34(6):741-4.
340. Nowell WR. International quarantine for control of mosquito-borne diseases on Guam. Aviation Space and Environmental Medicine. 1977;48(1):53-60.
341. Nozais JP. [New Guinea and Australia: their common pathology]. Bulletin de la Société de Pathologie Exotique et de ses Filiales. 1985;78(5):657-65.
342. Nye ER. The mosquito in the Pacific. Antenna. 1998;22(3):121-3.
343. O'Connor L, Plichart C, Sang AC, Brelsfoard CL, Bossin HC, Dobson SL. Open release of male mosquitoes infected with a *Wolbachia* biopesticide: field performance and infection containment. PLoS Neglected Tropical Diseases. 2012;6(11):e1797.
344. Ohta N. [Endemic tropical diseases: comtemporary health problem due to abandoned diseases in the developing world]. Kansenshogaku Zasshi. 2006;80(5):469-74.
345. Okamoto K, Nagayoshi H, Matsuyama M, Nagano T, Nagata K. [Chyluria in Japan--report of the present status]. Nihon Hinyokika Gakkai Zasshi. 1976;67(9):677-88.
346. Okamoto K, Ohi Y. Recent distribution and treatment of filarial chyluria in Japan. Journal of Urology. 1983;129(1):64-7.
347. Ottesen EA. Towards eliminating lymphatic filariasis; Chapter 8 in: ‘Lymphatic Filariasis’ ed Nutman TB. London: Imperial College Press; 2000:201-215. [Pasvol G, Hoffman SL (Series Editors): Tropical Medicine Science and Practice, vol 1.].
348. Ottesen EA, Duke BOL, Karam M, Behbehani K. Strategies and tools for the control/elimination of lymphatic filariasis. Bulletin of the World Health Organization. 1997;75(6):491-503.
349. Ottesen EA, Weil GJ, Lammie PJ, Bradley MH, Kumaraswami V, Addiss DG, et al. Towards a strategic plan for research to support the global program to eliminate lymphatic filariasis - Summary of immediate needs and opportunities for research on lymphatic filariasis identified by the filariasis community of scientists in association with an "LF research forum", convened in Philadelphia, December 9-10, 2003. American Journal of Tropical Medicine and Hygiene. 2004;71(5):1-46.
350. Ottesen EA, Weller PF, Lunde MN, Hussain R. Endemic filariasis on a Pacific Island .2. Immunological aspects - immunoglobulin, complement, and specific antifilarial IgG, IgM and IgE antibodies. American Journal of Tropical Medicine and Hygiene. 1982;31(5):953-61.
351. Outin-Fabre D, Saugrain J, Stanghellini A, Pichon G. [Pilot study of a campaign against filariasis in an insular environment (Moorea, French Polynesia)]. Bulletin of the World Health Organization. 1972;46(2):253-6.
352. Oyamada Y, Funae O, Kamegaya Y, Soejima K, Nakamura H, Mori S, et al. [A case of tropical eosinophilia associated with pleural effusion]. Nihon Kyobu Shikkan Gakkai Zasshi. 1995;33(4):451-5.
353. Palmer K, Ichimori K. Strategies for the control of intestinal helminths and lymphatic filariasis: initiatives in the WHO Western Pacific Region. In: Crompton DWT, Montresor A, Nesheim MC, Savioli L, editors. Controlling Disease Due to Helminth Infections. Geneva: WHO; 2003. p. 75-9.
354. Park CB. Microfilaria density distribution in the human population and its infectivity index for the mosquito population. Parasitology. 1988;96 (Pt 2):265-71.
355. Perolat P, Chanteau S, Roux J. [Value of a papain-treated homologous microfilarial antigen in the diagnosis of filariasis due to *Wuchereria bancrofti* var. *pacifica*. Analysis of 237 cases]. Médecine Tropicale : Revue du Corps de Santé Colonial. 1986;46(2):169-72.
356. Perolat P, Guidi C, Riviere F, Roux J. [Bancroftian filariasis in French Polynesia. Epidemiologic status and perspectives after a 35-year preventive campaign]. Bulletin de la Société de Pathologie Exotique et de ses Filiales. 1986;79(1):78-88.
357. Pichon G. [Migrations of microfilariae and Oceanic peoples. An approach to speciation in Bancrofti and Malayan filariae, by the study of the periodicity of microfilariae as a function of density; contribution to the prehistory of the Pacific area]. Annales de Parasitologie Humaine et Annales de parasitologie humaine et comparée. 1981;56(1):107-20.
358. Plichart C, Legrand AM. Detection and characterization of *Wolbachia* infections in *Wuchereria bancrofti* (Spirurida: Onchocercidae) var. *pacifica* and *Aedes (Stegomyia) polynesiensis* (Diptera: Culicidae). American Journal of Tropical Medicine and Hygiene. 2005;73(2):354-8.
359. Plichart C, Lemoine A. Monitoring and evaluation of lymphatic filariasis interventions: an improved PCR-based pool screening method for high throughput *Wuchereria bancrofti* detection using dried blood spots. Parasites and Vectors. 2013;6.
360. Plichart C, Sechan Y, Davies N, Legrand AM. PCR and dissection as tools to monitor filarial infection of *Aedes polynesiensis* mosquitoes in French Polynesia. Filaria Journal. 2006;5:2.
361. Pretrick M, Melrose W, Chaine JP, Canyon D, Carron J, Graves PM, et al. Identification and control of an isolated, but intense focus of lymphatic filariasis on Satawal Island, Federated States of Micronesia, in 2003. Tropical Medicine and Health. 2017;45:17.
362. Prybylski D, Alto WA, Mengeap S, Odaibaiyue S. Introduction of an integrated community-based bancroftian filariasis control program into the Mt-Bosavi region of the Southern Highlands of Papua New Guinea. Papua New Guinea Medical Journal. 1994;37(2):82-9.
363. Raccurt CP. [The persistence of *Wuchereria bancrofti* in Ouvea Island (New Caledonia)]. Médecine Tropicale : Revue du Corps de Santé Colonial. 1999;59(3):311.
364. Rajan TV. Neonatal tolerance and patent filarial infection. Trends in Parasitology. 2007;23(10):459-62.
365. Rajendran R, Sunish IP, Mani TR. Mass treatment of filariasis in New Guinea. New England Journal of Medicine. 2003;348(12):1180-.
366. Rakai IM, Naserua JD, Macnamara FN, Pillai JS. Mosquito-borne infections in Fiji. 4. Biting times for village mosquitoes and human filaria transmission potential of *Aedes polynesiensis* and *Aedes pseudoscutellaris*. Journal of Medical Entomology. 1974;11(5):588-94.
367. Ramaiah KD. Population migration: implications for lymphatic filariasis elimination programmes. PLoS Neglected Tropical Diseases. 2013;7(3).
368. Ramaiah KD, Ottesen EA. Progress and impact of 13 years of the Global Programme to Eliminate Lymphatic Filariasis on reducing the burden of filarial disease. PLoS Neglected Tropical Diseases. 2014;8(11).
369. Ramalingam S. A survey of mosquitoes and mosquito-borne diseases in some Central and South Pacific islands. Tropical Biomedicine. 1984;1(2):133-44.
370. Ramesh A, Small ST, Kloos ZA, Kazura JW, Nutman TB, Serre D, et al. The complete mitochondrial genome sequence of the filarial nematode *Wuchereria bancrofti* from three geographic isolates provides evidence of complex demographic history. Molecular and Biochemical Parasitology. 2012;183(1):32-41.
371. Rao RU, Atkinson LJ, Ramzy RMR, Helmy H, Farid HA, Bockarie MJ, et al. A real-time PCR-based assay for detection of *Wuchereria bancrofti* DNA in blood and mosquitoes. American Journal of Tropical Medicine and Hygiene. 2006;74(5):826-32.
372. Rao RU, Huang YF, Bockarie MJ, Susapu M, Laney SJ, Weil GJ. A qPCR-based multiplex assay for the detection of *Wuchereria bancrofti*, *Plasmodium falciparum* and *Plasmodium* vivax DNA. Transactions of the Royal Society of Tropical Medicine and Hygiene. 2009;103(4):365-70.
373. Rebollo MP, Bockarie MJ. Toward the elimination of lymphatic filariasis by 2020: treatment update and impact assessment for the endgame. Expert Review of Anti-Infective Therapy. 2013;11(7):723-31.
374. Rebollo MP, Bockarie MJ. Shrinking the lymphatic filariasis map: update on diagnostic tools for mapping and transmission monitoring. Parasitology. 2014;141(14):1912-7.
375. Ree HI, Mape J, Yaviong J. Studies on *Anopheles farauti* in the Republic of Vanuatu. Yonsei Reports on Tropical Medicine. 1991;22:43-54.
376. Reeder JC. Health research in Papua New Guinea. Trends in Parasitology. 2003;19(6):241-5.
377. Reeve D, Melrose W. Evaluation of the Og34C filter paper technique in lymphatic filariasis prevalence studies. Lymphology. 2014;47(2):65-72.
378. Reid EC, Kimura E. Microfilaria prevalence of diurnally subperiodic *Wuchereria bancrofti* among people having a medical checkup in American Samoa in the past 17 years. Journal of Tropical Medicine and Hygiene. 1993;96(2):118-23.
379. Reiff DM, Kaneko A, Taleo G, Amos M, Lum JK. Population structure and gene flow of *Anopheles farauti* s.s. (Diptera: Culicidae) among ten sites on five islands of Vanuatu: implications for malaria control. Journal of Medical Entomology. 2007;44(4):601-7.
380. Reimer LJ, Thomsen EK, Tisch DJ, Henry-Halldin CN, Zimmerman PA, Baea ME, et al. Insecticidal bed nets and filariasis transmission in Papua New Guinea. New England Journal of Medicine. 2013;369(8):745-53.
381. Rivière F, Klein JM, Duval J, Sechan Y, Thirel R, Faaruia M, et al. Ecology of *Aedes (Stegomyia) polynesiensis* Marks, 1951 (Diptera: Culicidae), mosquito vector of bancroft's filariasis. II. Studies in land crab holes of *Cardisoma carnifex* Herbst. Annales de la Société Entomologique de France. 1998;34(3):259-83.
382. Rivière F, Klein JM, Thirel R, Chebret M. Ecology of *Aedes (Stegomyia) polynesiensis* Marks, 1951 (Diptera : Culicidae), vector of bancroft's filariasis. I. Studies in coconuts eaten by rats as breeding sites. Annales de la Société Entomologique de France. 1998;34(2):195-207.
383. Rodhain F, Gaxotte P, Boutonnier A. [Results of an entomological survey of mosquitoes (Diptera: Culicidae) in Hermit Islands and Wuvulu Island (Papua, New Guinea)]. Bulletin de la Société de Pathologie Exotique et de ses Filiales. 1980;73(2):214-9.
384. Rodhain F, Rodhainrebourg F. Geographical distribution of lymphatic filariasis. 6. Northeastern Asia and Western Pacific Islands. Médecine et Maladies Infectieuses. 1976;6(12):506-13.
385. Rodhain F, Rodhainrebourg F. Geographical distribution of lymphatic filariasis. 7.South-Pacific. Médecine et Maladies Infectieuses. 1977;7(4):217-24.
386. Rosen L, Rozeboom LE, Reeves WC, Saugrain J, Gubler DJ. A field trial of competitive displacement of *Aedes polynesiensis* by *Aedes albopictus* on a Pacific atoll. American Journal of Tropical Medicine and Hygiene. 1976;25(6):906-13.
387. Roux J, Perolat P, Cartel JL, Boutin JP, Sechan Y, Larivière M, et al. [A study of ivermectin in the treatment of lymphatic filariasis due to *Wuchereria bancrofti* var. *pacifica* in French Polynesia]. Bulletin de la Société de Pathologie Exotique et de ses Filiales. 1989;82(1):72-81.
388. Rozendaal JA. Impregnated mosquito nets and curtains for self-protection and vector control. Tropical Diseases Bulletin. 1989;86(7):1-41.
389. Russell RC. The relative attractiveness of carbon dioxide and octenol in CDC- and EVS-type light traps for sampling the mosquitoes *Aedes aegypti* (L.), *Aedes polynesiensis* Marks, and *Culex quinquefasciatus* say in Moorea, French Polynesia. Journal of Vector Ecology. 2004;29(2):309-14.
390. Russell RC, Ritchie SA. Surveillance and behavioral investigations of *Aedes aegypti* and *Aedes polynesiensis* in Moorea, French Polynesia, using a sticky ovitrap. Journal of the American Mosquito Control Association. 2004;20(4):370-5.
391. Russell RC, Webb CE, Davies N. *Aedes aegypti* (L.) and *Aedes polynesiensis* Marks (Diptera: Culicidae) in Moorea, French Polynesia: a study of adult population structures and pathogen (*Wuchereria bancrofti* and *Dirofilaria immitis*) infection rates to indicate regional and seasonal epidemiological risk for dengue and filariasis. Journal of Medical Entomology. 2005;42(6):1045-56.
392. Sakakura T, Ikeuchi T, Watanabe H. [Chyluria treated with stripping of the renal pedicle: a case report]. Hinyokika Kiyo. 1996;42(3):221-3.
393. Sakugawa H, Kadena K, Oyakawa T, Kuniyoshi T, Akamine K, Uehara T, et al. [The epidemiological study on hepatitis B virus infection in Miyako district--relationship to the mass survey of filariasis]. Kansenshogaku Zasshi. 1990;64(7):774-80.
394. Salfield S. Filarial arthritis in the Sepik District of Papua New Guinea. Medical Journal of Australia. 1975;1(9):264-7.
395. Samarawickrema WA, Kimura E, Sones F, Paulson GS, Cummings RF. Natural infections of *Dirofilaria Immitis* in *Aedes (Stegomyia) polynesiensis* and *Aedes (Finlaya) samoanus* and their implication in human health in Samoa. Transactions of the Royal Society of Tropical Medicine and Hygiene. 1992;86(2):187-8.
396. Samarawickrema WA, Kimura E, Spears GF, Penaia L, Sone F, Paulson GS, et al. Distribution of vectors, transmission indices and microfilaria rates of subperiodic *Wuchereria bancrofti* in relation to village ecotypes in Samoa. Transactions of the Royal Society of Tropical Medicine and Hygiene. 1987;81(1):129-35.
397. Samarawickrema WA, Parkinson AD, Kere N, Galo O. Seasonal abundance and biting behaviour of *Anopheles punctulatus* and *An. koliensis* in Malaita Province, Solomon Islands, and a trial of permethrin impregnated bed nets against malaria transmission. Medical and Veterinary Entomology. 1992;6(4):371-8.
398. Samarawickrema WA, Sone F, Cummings RF. Natural infection of *Wuchereria bancrofti* in *Aedes (Stegomyia) polynesiensis* and *Aedes (Finlaya) samoanus* in Samoa. Transactions of the Royal Society of Tropical Medicine and Hygiene. 1987;81(1):124-8.
399. Samarawickrema WA, Sone F, Cummings RF. Seasonal abundance, diel biting activity and parity of *Aedes polynesiensis* Marks and *A. samoanus* (Gruenberg) (Diptera: Culicidae) in Samoa. Bulletin of Entomological Research. 1987;77(2):191-200.
400. Samarawickrema WA, Sone F, Kimura E, Self LS, Cummings RF, Paulson GS. The relative importance and distribution of *Aedes polynesiensis* and *Ae. aegypti* larval habitats in Samoa. Medical and Veterinary Entomology. 1993;7(1):27-36.
401. Samarawickrema WA, Sone F, Paulson GS, Kimura E, Uchida K, Cummings RF. Observations on *Culex quinquefasciatus* Say in relation to transmission of filariasis due to subperiodic *Wuchereria bancrofti* in Samoa. Annals of Tropical Medicine and Parasitology. 1992;86(5):517-22.
402. Samarawickrema WA, Sone F, Self LS, Cummings RF, Paulson GS. Distribution of breeding and control of the filariasis vector *Aedes samoanus* in leaf axils of *Pandanus* in Samoa. Medical and Veterinary Entomology. 1992;6(4):367-70.
403. Samarawickrema WA, Spears GFS, Sone F, Ichimori K, Cummings RF. Filariasis transmission in Samoa. 2. Some factors related to the development of microfilariae in the intermediate host. Annals of Tropical Medicine and Parasitology. 1985;79(1):101-7.
404. Samarawickrema WA, Spears GFS, Sone F, Ichimori K, Cummings RF. Filariasis transmission in Samoa. 1. Relation between density of microfilariae and larval density in laboratory-bred and wild-caught *Aedes (Stegomyia) polynesiensis* (Marks) and wild-caught *Aedes (Finlaya) samoanus* (Gruenberg). Annals of Tropical Medicine and Parasitology. 1985;79(1):89-100.
405. Sapak P, Williams, G. The influence of bednets on bancroftian filariasis in Buhutu valley, Papua New Guinea. Pacific Health Dialog. 1997;4(1):35-8.
406. Sapak P. Can the Pacific get rid of filariasis by the year 2020? A conference report. Pacific Health Dialog. 1998;5(1):102-4.
407. Sapak P, Vallely A, Giurina P, Maibani C. Diurnal subperiodic bancroftian filariasis in Dogura, PNG. Pacific Health Dialog. 1998;5(1):38-40.
408. Sapak P, Williams G, Bryan J, Riley I. Efficacy of mass single-dose diethylcarbamazine and DEC-fortified salt against bancroftian filariasis in Papua New Guinea six months after treatment. Papua New Guinea Medical Journal. 2000;43(3-4):213-20.
409. Sasa M. Anti-filariasis campaign: its history and future prospects. Progress in drug research. Fortschritte der Arzneimittelforschung. Progrès des recherches pharmaceutiques. 1974;18:259-68.
410. Sasa M. Methods for estimating the efficiency of detection of microfilariae in various volumes of blood samples. Southeast Asian Journal of Tropical Medicine and Public Health. 1974;5(2):197-210.
411. Sasa M. Human Filariasis. A Global Survey of Epidemiology and Control. Baltimore. London. Tokyo: University Park Press; 1976.
412. Sasagawa E. Prevalence of lymphatic filariasis from 1999 through 2007 in Nauru, a set of solitary islands in the Southern Pacific. Tropical Medicine and Health. 2009;37(2):63-8.
413. Sato H, Otsuji Y, Maeda T. [Filariasis]. Naika. 1971;27(3):438-42.
414. Saugrain J, Outin-Fabre D. [Results of 20 years of control of subperiodic bancroft filariasis in French Polynesia]. Bulletin of the World Health Organization. 1972;46(2):249-52.
415. Schmaedick MA, Ball TS, Burkot TR, Gurr NE. Evaluation of three traps for sampling *Aedes polynesiensis* and other mosquito species in American Samoa. Journal of the American Mosquito Control Association. 2008;24(2):319-22.
416. Schmaedick MA, Koppel AL, Pilotte N, Torres M, Williams SA, Dobson SL, et al. Molecular xenomonitoring using mosquitoes to map lymphatic filariasis after mass drug administration in American Samoa. PLoS Neglected Tropical Diseases. 2014;8(8):e3087.
417. Schmidt ER, Foley DH, Bugoro H, Bryan JH. A morphological study of the *Anopheles punctulatus* group (Diptera: Culicidae) in the Solomon Islands, with a description of *Anopheles (Cellia) irenicus* Schmidt, sp.n. Bulletin of Entomological Research. 2003;93(6):515-26.
418. Schratz A, Pineda MF, Reforma LG, Fox NM, Le Anh T, Tommaso Cavalli-Sforza L, et al. Neglected diseases and ethnic minorities in the Western Pacific Region: exploring the links. Advances in Parasitology. 2010;72:79-107. Chapter 4 in: ‘Im[portant Helminth Infections in Southeast Asia: Diversity and Potential for Control and Elimination’, Part A](http://www.sciencedirect.com/science/journal/0065308X/72/supp/C) Edited by Xiao-Nong Zhou, RobertBergquist, RemigioOlveda,JürgUtzinger.
419. Schuurkamp GJ. The epidemiology of malaria and filariasis in the Ok Tedi region of Western Province, Papua New Guinea. PhD thesis, University of Papua New Guinea. Tabubil: Ok Tedi Mining Limited; 1992. 342 p.
420. Schuurkamp GJ, Kereu RK, Bulungol PK. Diethylcarbamazine in the control of bancroftian filariasis in the highly endemic Ok Tedi area of Papua New Guinea: phase 1. Papua New Guinea Medical Journal. 1990;33(2):89-98.
421. Schuurkamp GJ, Kereu RK, Bulungol PK, Kawereng A, Popon WH, Crane GG, et al. Diethylcarbamazine in the control of splenomegaly associated with bancroftian filariasis in the Ok Tedi area of Papua New Guinea. Transactions of the Royal Society of Tropical Medicine and Hygiene. 1992;86(5):531-6.
422. Schuurkamp GJ, Kereu RK, Bulungol PK, Kawereng A, Spicer PE. Diethylcarbamazine in the control of bancroftian filariasis in the Ok Tedi area of Papua New Guinea: phase 2 -- annual single-dose treatment. Papua New Guinea Medical Journal. 1994;37(2):65-81.
423. Schuurkamp GJT, Matango M, Kereu R, Napil J. Malaria, splenomegaly and filariasis in the Ok Tedi area of the Star Mountains, Papua New Guinea, three years after residual DDT spraying. Papua New Guinea Medical Journal. 1987;30(4):291-300.
424. Scrimgeour EM, Matz LR, Aaskov JG. A study of arthritis in Papua New Guinea. Australian and New Zealand Journal of Medicine. 1987;17(1):51-4.
425. Selve BP, Bwadua S, Misa M, James K, Usurup JP, Turner P, et al. Community empowerment in the control of lymphatic filariasis in Misima, Milne Bay Province using diethylcarbamazine in combination with albendazole. Papua New Guinea Medical Journal. 2000;43(3-4):183-7.
426. Sherman RH, Goldman LB, deVere White RW. Filarial chyluria as a cause of acute urinary retention. Urology. 1987;29(6):642-5.
427. Singh BK, Bockarie MJ, Gambhir M, Siba PM, Tisch DJ, Kazura J, et al. Sequential modelling of the effects of mass drug treatments on anopheline-mediated lymphatic filariasis infection in Papua New Guinea. PLoS One. 2013;8(6):e67004.
428. Sinka ME, Bangs MJ, Manguin S, Chareonviriyaphap T, Patil AP, Temperley WH, et al. The dominant *Anopheles* vectors of human malaria in the Asia-Pacific region: occurrence data, distribution maps and bionomic précis. Parasites and Vectors. 2011;4(1)(89).
429. Small ST, Ramesh A, Bun K, Reimer L, Thomsen E, Baea M, et al. Population genetics of the filarial worm *Wuchereria bancrofti* in a post-treatment region of Papua New Guinea: insights into diversity and life history. PLoS Neglected Tropical Diseases. 2013;7(7):e2308.
430. Small ST, Reimer LJ, Tisch DJ, King CL, Christensen BM, Siba PM, et al. Population genomics of the filarial nematode parasite *Wuchereria bancrofti* from mosquitoes. Molecular Ecology. 2016;25(7):1465-77.
431. Small ST, Tisch DJ, Zimmerman PA. Molecular epidemiology, phylogeny and evolution of the filarial nematode *Wuchereria bancrofti*. Infection, Genetics and Evolution. 2014;28:33-43.
432. Snow LC, Bockarie MJ, Michael E. Transmission dynamics of lymphatic filariasis: vector-specific density dependence in the development of *Wuchereria bancrofti* infective larvae in mosquitoes. Medical and Veterinary Entomology. 2006;20(3):261-72.
433. Snow LC, Michael E. Transmission dynamics of lymphatic filariasis: density-dependence in the uptake of *Wuchereria bancrofti* microfilariae by vector mosquitoes. Medical and Veterinary Entomology. 2002;16(4):409-23.
434. Southgate BA. Studies of filariasis in the Pacific. 1. A field trial of a counting-chamber technique for the determination of microfilarial rates and densities. Southeast Asian Journal of Tropical Medicine and Public Health. 1973;4(2):172-8.
435. Southgate BA. Community diagnosis and management in bancroftian filariasis. Tropical Doctor. 1974;4(1):3-5.
436. Southgate BA. The significance of low-density microfilaremia in the transmission of lymphatic filarial parasites. Journal of Tropical Medicine and Hygiene. 1992;95(2):79-86.
437. Southgate BA. Intensity and efficiency of transmission and the development of microfilaraemia and disease: their relationship in lymphatic filariasis. Journal of Tropical Medicine and Hygiene. 1992;95(1):1-12.
438. Southgate BA, Bryan JH. Factors affecting transmission of *Wuchereria bancrofti* by anopheline mosquitoes. 4. Facilitation, limitation, proportionality and their epidemiological significance. Transactions of the Royal Society of Tropical Medicine and Hygiene. 1992;86(5):523-30.
439. Sprent JF. Parasitology in Brisbane. International Journal for Parasitology. 1986;16(4):281-5.
440. Steel C, Guinea A, Mccarthy JS, Ottesen EA. Long-term effect of prenatal exposure to maternal microfilaremia on immune responsiveness to filarial parasite antigens. Lancet. 1994;343(8902):890-3.
441. Steel C, Guinea A, Ottesen EA. Evidence for protective immunity to bancroftian filariasis in the Cook Islands. Journal of Infectious Diseases. 1996;174(3):598-605.
442. Steel C, Kubofcik J, Ottesen EA, Nutman TB. Antibody to the filarial antigen Wb123 reflects reduced transmission and decreased exposure in children born following single mass drug administration (MDA). PLoS Neglected Tropical Diseases. 2012;6(12):e1940.
443. Steel C, Nutman TB. Altered T cell memory and effector cell development in chronic lymphatic filarial infection that is independent of persistent parasite antigen. PLoS One. 2011;6(4):e19197.
444. Steel C, Ottesen EA. Evolution of immunologic responsiveness of persons living in an area of endemic bancroftian filariasis: a 17-year follow-up. Journal of Infectious Diseases. 2001;184(1):73-9.
445. Steel C, Ottesen EA, Weller PF, Nutman TB. Worm burden and host responsiveness in *Wuchereria bancrofti* infection: use of antigen detection to refine earlier assessments from the South Pacific. American Journal of Tropical Medicine and Hygiene. 2001;65(5):498-503.
446. Stemmermann GN. Patterns of disease among Japanese living in Hawaii. Archives of Environmental Health. 1970;20(2):266-73.
447. Stone CM, Lindsay SW, Chitnis N. How effective is integrated vector management against malaria and lymphatic filariasis where the diseases are transmitted by the same vector? PLoS Neglected Tropical Diseases. 2014;8(12).
448. Sung RTM, Bessenay J, Mojon M, Garin JP. Lymphatic filariasis in the Wallis and Futuna Islands. Clinical, biological and chemoprophylactic data. Lyon Medical. 1979;241(6):339-45.
449. Suzuki R, Morita H, Sugenoya Y, Mizobuchi M, Yamamoto W, Ideura T, et al. [A case report of chronic chyluria probably due to bancroftian filariasis, which showed hypoproteinemia]. Nihon Jinzo Gakkai Shi. 2001;43(2):63-8.
450. Suzuki T, Sone F. The bionomics of filariasis vectors in Western Samoa. Japanese Journal of Sanitary Zoology. 1974;25(3):251-7.
451. Suzuki T, Sone F. Filarial infection in vector mosquitos after mass drug administration in Western Samoa. Tropical Medicine. 1975;16(3):147-56.
452. Tada I. Filariasis control with diethylcarbamazine in three major endemic areas in Japan. Tropical Medicine and Health. 2011;39(1 Suppl 2):21-3.
453. Tada I. Lymphatic filariasis and its control in Japan -the background of success. Tropical Medicine and Health. 2011;39(1 Suppl 2):15-20.
454. Tada I, Otsuji Y, Harada R, Mimori T, Fukumoto H. Skin test study of bancroftian filariasis in Kuroshima Island, Okinawa: a 13-year longitudinal study during a control campaign. American Journal of Tropical Medicine and Hygiene. 1982;31(5):962-7.
455. Tajima K, Fujita K, Tsukidate S, Oda T, Tominaga S, Suchi T, et al. Seroepidemiological studies on the effects of filarial parasites on infestation of adult T-cell leukemia virus in the Goto Islands, Japan. GANN Japanese Journal of Cancer Research. 1983;74(2):188-91.
456. Tajima K, Tominaga S, Shimizu H, Suchi T. A hypothesis on the etiology of adult T-cell leukemia/lymphoma. GANN Japanese Journal of Cancer Research. 1981;72(5):684-91.
457. Taleo F, Taleo G, Graves PM, Wood P, Kim SH, Ozaki M, et al. Surveillance efforts after mass drug administration to validate elimination of lymphatic filariasis as a public health problem in Vanuatu. Tropical Medicine and Health. 2017;45:18.
458. Tambo E, Ai L, Zhou X, Chen JH, Hu W, Bergquist R, et al. Surveillance-response systems: the key to elimination of tropical diseases. Infectious Diseases of Poverty. 2014;3:17.
459. Tanaka S, Tsuruya K, Tsuchimoto A, Eriguchi M, Kitazono T. Successful treatment of massive proteinuria and severe chyluria by inhibition of cholesterol absorption with ezetimibe in a patient with filariasis. Clinical Kidney Journal. 2012;5(5):449-52.
460. Taylor B. Imagos of mosquitoes collected in Vanuatu,1987 identification and comments. Rivista di Parassitologia. 1989;6:13-5.
461. Taylor B. Mosquitoes and man in the Pacific - more than just words. Antenna. 2000;22(1):201-4.
462. Taylor MJ, Hoerauf A, Bockarie M. Lymphatic filariasis and onchocerciasis. Lancet. 2010;376(9747):1175-85.
463. Terashi H, Tahara S, Shibuya H, Sumino Y, Hirai K, Ohno H, et al. Treatment of filarial chyluria with lymphovenous shunt anastomosis: report of three cases in Japan. Plastic and Reconstructive Surgery. 2003;112(4):1049-53.
464. Thomsen EK, Sanuku N, Baea M, Satofan S, Maki E, Lombore B, et al. Efficacy, safety, and pharmacokinetics of coadministered diethylcarbamazine, albendazole, and ivermectin for treatment of bancroftian filariasis. Clinical Infectious Diseases. 2016;62(3):334-41.
465. Tisch DJ, Alexander ND, Kastens W, Bockarie MJ, Kazura JW. Longitudinal analysis of acute lymphatic filariasis in Papua New Guinea: Evaluation of annual mass drug administration on disease. American Journal of Tropical Medicine and Hygiene. 2005;73(6):175-6.
466. Tisch DJ, Alexander NDE, Kiniboro B, Dagoro H, Siba PM, Bockarie MJ, et al. Reduction in acute filariasis morbidity during a mass drug administration trial to eliminate lymphatic filariasis in Papua New Guinea. PLoS Neglected Tropical Diseases. 2011;5(7):e1241.
467. Tisch DJ, Bockarie MJ, Dimber Z, Kiniboro B, Tarongka N, Hazlett FE, et al. Mass drug administration trial to eliminate lymphatic filariasis in Papua New Guinea: changes in microfilaremia, filarial antigen, and Bm14 antibody after cessation. American Journal of Tropical Medicine and Hygiene. 2008;78(2):289-93.
468. Tisch DJ, Hazlett FE, Bockarie MJ, Kastens W, Kazura JW. Long term effect of mass drug administration on the presence of antibodies against Bm14 as a measure of lymphatic filariasis exposure and infection in Papua New Guinea. American Journal of Tropical Medicine and Hygiene. 2006;75(5):100-1.
469. Tisch DJ, Hazlett FE, Kastens W, Alpers MP, Bockarie MJ, Kazura JW. Ecologic and biologic determinants of filarial antigenemia in bancroftian filariasis in Papua New Guinea. Journal of Infectious Diseases. 2001;184(7):898-904.
470. Tisch DJ, Kastens W, Moses BJ, Baisor M, Susapa M, Sepe D, et al. Transmission intensity and biomarkers of *Wuchereria bancrofti* infection 10 years after cessation of mass drug administration to eliminate lymphatic filariasis in Papua New Guinea. American Journal of Tropical Medicine and Hygiene. 2009;81(5):294-.
471. Tisch DJ, Kazura JW. Mass treatment of filariasis in New Guinea - Reply.New England Journal of Medicine. 2003;348(12):1180-1.
472. Tisch DJ, Michael E, Kazura JW. Mass chemotherapy options to control lymphatic filariasis: a systematic review. Lancet Infectious Diseases. 2005;5(8):514-23.
473. Tisch DJ, Salvana E, Kastens W, Bockarie M, Alexander N, Kazura JW. Familial aggregation of acute lymphatic filariasis in Papua New Guinea. American Journal of Tropical Medicine and Hygiene. 2007;77(5):103-.
474. Tobian AAR, Tarongka N, Baisor M, Bockarie M, Kazura JW, King CL. Sensitivity and specificity of ultrasound detection and risk factors for filarial-associated hydroceles. American Journal of Tropical Medicine and Hygiene. 2003;68(6):638-42.
475. Tuicakau MS, Kama M. Filariasis in Fiji. Australasian Journal of Dermatology. 2012;53:12-.
476. Turner P, Copeman B, Gerisi D, Speare R. A comparison of the Og4C3 antigen capture ELISA, the Knott test, an IgG4 assay and clinical signs, in the diagnosis of bancroftian filariasis. Tropical Medicine and Parasitology. 1993;44(1):45-8.
477. Turner PF. A study of the CD4: CD8 ratio in peripheral T lymphocytes of rural Papua New Guineans: a reduced ratio assessed with regard to infectious agents. Papua New Guinea Medical Journal. 1993;36(3):210-4.
478. Turner PF, Rockett KA, Ottesen EA, Francis H, Awadzi K, Clark IA. Interleukin-6 and tumor necrosis factor in the pathogenesis of adverse reactions after treatment of lymphatic filariasis and onchocerciasis. Journal of Infectious Diseases. 1994;169(5):1071-5.
479. Turner PF, Usurup JP. A possible case of lymphatic filariasis in a white miner in Papua New Guinea. The Medical Journal of Australia. 1997;166(4):223.
480. Tuten HC, Stone CM, Dobson SL. Swarming behavior of *Aedes polynesiensis* (Diptera: Culicidae) and characterization of swarm markers in American Samoa. Journal of Medical Entomology. 2013;50(4):740-7.
481. Uchida K, Ichimori K, Paulson GS, Cox PA, Sone F, Samarawickrema WA. Laboratory studies of *Ochlerotatus samoanus* in association with leaf axils of *Freycinetia* (Pandanaceae) in Samoa. Journal of the American Mosquito Control Association. 2002;18(2):81-5.
482. Urbani C, Palmer K. Drug-based helminth control in Western Pacific countries: a general perspective. Tropical Medicine and International Health.. 2001;6(11):935-44.
483. Vigneron E. The epidemiological transition in an overseas territory: disease mapping in French Polynesia. Social Science and Medicine. 1989;29(8):913-22.
484. Wada Y. Vector mosquitoes of filariasis in Japan. Tropical Medicine and Health. 2011;39(1 Suppl 2):39-45.
485. Wada Y, Kimura E, Takagi M, Tsuda Y. Facilitation in *Anopheles* and spontaneous disappearance of filariasis: has the concept been verified with sufficient evidence? Tropical Medicine and Parasitology. 1995;46(1):27-30.
486. Webber RH. Vector control of filariasis in the Solomon Islands. Southeast Asian Journal of Tropical Medicine and Public Health. 1975;6(3):430-4.
487. Webber RH. Theoretical considerations in the vector control of filariasis. Southeast Asian Journal of Tropical Medicine and Public Health. 1975;6(4):544-8.
488. Webber RH. The natural decline of *Wuchereria bancrofti* infection in a vector control situation in the Solomon Islands. Transactions of the Royal Society of Tropical Medicine and Hygiene. 1977;71(5):396-400.
489. Webber RH. Eradication of *Wuchereria bancrofti* infection through vector control. Transactions of the Royal Society of Tropical Medicine and Hygiene. 1979;73(6):722-4.
490. Webber RH. Can anopheline-transmitted filariasis be eradicated? Journal of Tropical Medicine and Hygiene. 1991;94(4):241-4.
491. Webber RH, Southgate BA. The maximum density of anopheline mosquitoes that can be permitted in the absence of continuing transmission of filariasis. Transactions of the Royal Society of Tropical Medicine and Hygiene. 1981;75(4):499-506.
492. Weil GJ, Curtis KC, Fischer PU, Won KY, Lammie PJ, Joseph H, et al. A multicenter evaluation of a new antibody test kit for lymphatic filariasis employing recombinant *Brugia malayi* antigen Bm-14. Acta Tropica. 2011;120 Suppl 1:S19-22.
493. Weil GJ, Kastens W, Susapu M, Laney SJ, Williams SA, King CL, et al. The impact of repeated rounds of mass drug administration with diethylcarbamazine plus albendazole on bancroftian filariasis in Papua New Guinea. PLoS Neglected Tropical Diseases. 2008;2(12):e344.
494. Welch JS, Dobson C. The prevalence of antibodies to *Dirofilaria immitis* in aboriginal and caucasian Australians. Transactions of the Royal Society of Tropical Medicine and Hygiene. 1974;68(6):466-72.
495. Weller PF, Ottesen EA. Failure of diethylcarbamazine as a provocative test in subperiodic *Wuchereria bancrofti* filariasis. Transactions of the Royal Society of Tropical Medicine and Hygiene. 1978;72(1):31-2.
496. Weller PF, Ottesen EA, Heck L. Immediate and delayed hypersensitivity skin test responses to the *Dirofilaria immitis* filarial skin test (Sawada) antigen in *Wuchereria bancrofti* filariasis. American Journal of Tropical Medicine and Hygiene. 1980;29(5):809-14.
497. Weller PF, Ottesen EA, Heck L, Tere T, Neva FA. Endemic filariasis on a Pacific island. 1. Clinical, epidemiologic, and parasitologic aspects. American Journal of Tropical Medicine and Hygiene. 1982;31(5):942-52.
498. WHO. WHO expert committee on filariasis third report.. Geneva; 1974. World Health Organization technical report series No.542.
499. WHO. Intervention research on onchocerciasis and lymphatic filariasis. The Weekly Epidemiological Record. 2000;75(30):246-8.
500. WHO. Lymphatic filariasis. The Weekly Epidemiological Record. 2000;75(25):206-8.
501. WHO. Lymphatic filariasis. The Weekly Epidemiological Record. 2001;76(20):149-54.
502. WHO. Lymphatic filariasis: Progress report on mass drug administration in 2001. The Weekly Epidemiological Record. 2002;77(16):125-32.
503. WHO. Report on active surveillance for adverse events following the use of drug co-administrations in the global programme to eliminate lymphatic filariasis. The Weekly Epidemiological Record. 2003;78(36):315-7.
504. WHO. Lymphatic filariasis. The Weekly Epidemiological Record. 2003;78(20):171-9.
505. WHO. Report on the mid-term assessment of microfilaraemia reduction in sentinel sites of 13 countries of the Global Programme to Eliminate Lymphatic Filariasis. The Weekly Epidemiological Record. 2004;79(40):358-65.
506. WHO. Lymphatic filariasis: progress of disability prevention activities. The Weekly Epidemiological Record. 2004;79(47):417-24.
507. WHO. The PacELF Way: Towards the elimination of lymphatic filariasis from the Pacific, 1999-2005. Manila: WHO Western Pacific Region; 2006.
508. WHO. Managing morbidity and preventing disability in the Global Programme to Eliminate Lymphatic Filariasis: WHO position statement. The Weekly Epidemiological Record. 2011;86(51-52):581-5.
509. WHO. WHO position statement on integrated vector management to control malaria and lymphatic filariasis. The Weekly Epidemiological Record. 2011;86(13):121-7.
510. WHO. Global Programme to eliminate lymphatic filariasis: progress report on mass drug administration, 2010. The Weekly Epidemiological Record. 2011;86(35):377-88.
511. WHO. Transmission assessment surveys in the Global Programme to Eliminate Lymphatic Filariasis: WHO position statement. The Weekly Epidemiological Record. 2012;87(48):478-82.
512. WHO. Global programme to eliminate lymphatic filariasis: progress report, 2011. The Weekly Epidemiological Record. 2012;87(37):346-56.
513. WHO. Global programme to eliminate lymphatic filariasis: progress report, 2012. The Weekly Epidemiological Record. 2013;88(37):389-99.
514. WHO. Global programme to eliminate lymphatic filariasis: progress report, 2013. The Weekly Epidemiological Record. 2014;89(38):409-18.
515. WHO. Global programme to eliminate lymphatic filariasis: progress report, 2014. The Weekly Epidemiological Record. 2015;90(38):489-504.
516. WHO. Global programme to eliminate lymphatic filariasis: progress report, 2015. The Weekly Epidemiological Record. 2016;91(39):441-55.
517. WHO Western Pacific Region. Regional Action Plan for Neglected Tropical Diseases in the Western Pacific Region (2012-2016). Western Pacific. WHO Western Pacific Region; 2013.
518. Williams SA, Nicolas L, Lizotte-Waniewski M, Plichart C, Luquiaud P, Nguyen LN, et al. A polymerase chain reaction assay for the detection of *Wuchereria bancrofti* in blood samples from French Polynesia. Transactions of the Royal Society of Tropical Medicine and Hygiene. 1996;90(4):384-7.
519. Wilson AL, Dhiman RC, Kitron U, Scott TW, van den Berg H, Lindsay SW. Benefit of insecticide-treated nets, curtains and screening on vector borne diseases, excluding malaria: a systematic review and meta-analysis. PLoS Neglected Tropical Diseases. 2014;8(10).
520. Witt C, Ottesen EA. Lymphatic filariasis: an infection of childhood. Tropical Medicine and International Health. 2001;6(8):582-606.
521. Wynd S, Carron J, Selve B, Leggat PA, Melrose W, Durrheim DN. Qualitative analysis of the impact of a lymphatic filariasis elimination programme using mass drug administration on Misima Island, Papua New Guinea. Filaria Journal. 2007;6:1.
522. Wynd S, Durrheim DN, Carron J, Selve B, Chaine JP, Leggat PA, et al. Socio-cultural insights and lymphatic filariasis control--lessons from the Pacific. Filaria Journal. 2007;6:3.
523. Wynd S, Melrose WD, Durrheim DN, Carron J, Gyapong M. Understanding the community impact of lymphatic filariasis: a review of the sociocultural literature. Bulletin of the World Health Organization. 2007;85:493-8.
524. Yagi S, Goto T, Kawamoto K, Miyawaki I, Tanaka I, Mori K, et al. Endoscopic treatment of refractory filarial chyluria: a preliminary report. Journal of Urology. 1998;159(5):1615-8.
525. Yamada H, Hirabayashi F, Brunger C. [International Partnership for Therapeutic Drug Development of NTDs by DNDi]. YAKUGAKU ZASSHI. 2016;136(2):213-22.
526. Yamauchi S. A correlative study of hematuria observed in Hawaii caused by lymphatic filariasis and other urological conditions. International Congress Series. 1990;887:395-6.
527. Yong MK, Marshall CL, Eisen DP. Tropical pulmonary eosinophilia: a rare cause of cough in immigrants to Australia. Medical Journal of Australia. 2007;187(7):416-8.
528. Yoshida C. Filariasis control in Okinawa. Tropical Medicine and Health. 2011;39(1 Suppl 2):29-37.
529. Zahar AR, King M, Chow CY. A review and an annotated bibliography on subperiodic bancroftian filariasis with special reference to its vectors in Polynesia, South Pacific. WHO, Manila 1980. 492p.
530. Zeldenryk L, Gordon S, Gray M, Speare R, Melrose W. Disability measurement for lymphatic filariasis: a review of generic tools used within morbidity management programs. PLoS Neglected Tropical Diseases. 2012;6(9):e1768.
531. Zeldenryk LM, Gray M, Speare R, Gordon S, Melrose W. The emerging story of disability associated with lymphatic filariasis: a critical review. PLoS Neglected Tropical Diseases. 2011;5(12):e1366.
532. Zhong M, McCarthy J, Bierwert L, Lizotte-Waniewski M, Chanteau S, Nutman TB, et al. A polymerase chain reaction assay for detection of the parasite *Wuchereria bancrofti* in human blood samples. American Journal of Tropical Medicine and Hygiene. 1996;54(4):357-63.
